# AI-Assisted vs Unassisted Identification of Prostate Cancer in Magnetic Resonance Images

**DOI:** 10.1001/jamanetworkopen.2025.15672

**Published:** 2025-06-13

**Authors:** Jasper J. Twilt, Anindo Saha, Joeran S. Bosma, Anwar R. Padhani, David Bonekamp, Gianluca Giannarini, Roderick van den Bergh, Veeru Kasivisvanathan, Nancy Obuchowski, Derya Yakar, Mattijs Elschot, Jeroen Veltman, Jurgen Fütterer, Henkjan Huisman, Maarten de Rooij

**Affiliations:** 1 Minimally Invasive Image-Guided Intervention Center, Department of Medical Imaging, Radboud University Medical Center, Nijmegen, the Netherlands; 2 Diagnostic Image Analysis Group, Department of Medical Imaging, Radboud University Medical Center, Nijmegen, the Netherlands; 3 Paul Strickland Scanner Centre, Mount Vernon Cancer Centre, Northwood, United Kingdom; 4 Division of Radiology, Deutsches Krebsforschungszentrum, Heidelberg, Germany; 5 Urology Unit, Santa Maria della Misericordia University Hospital, Udine, Italy; 6 Department of Urology, Erasmus Medical Center, Rotterdam, the Netherlands; 7 Centre for Urology Imaging, Prostate, AI and Surgical Studies (COMPASS) Research Group, Division of Surgery and Interventional Sciences, University College London, London, United Kingdom; 8 Department of Quantitative Health Sciences, Cleveland Clinic Foundation, Cleveland, Ohio; 9 Department of Diagnostic Radiology, Cleveland Clinic Foundation, Cleveland, Ohio; 10 Department of Radiology, University Medical Center Groningen, Groningen, the Netherlands; 11 Department of Radiology, Netherlands Cancer Institute, Amsterdam, the Netherlands; 12 Department of Circulation and Medical Imaging, Norwegian University of Science and Technology, Trondheim, Norway; 13 Department of Radiology and Nuclear Medicine, St Olavs Hospital, Trondheim University Hospital, Trondheim, Norway; 14 Department of Radiology, Ziekenhuisgroep Twente, Hengelo, the Netherlands; 15 Department of Multi-Modality Medical Imaging, Technical Medical Centre, University of Twente, Enschede, the Netherlands; 16 Department of Medical Imaging, Radboud University Medical Center, Nijmegen, the Netherlands

## Abstract

**Importance:**

Artificial intelligence (AI) assistance in magnetic resonance imaging (MRI) assessment for prostate cancer shows promise for improving diagnostic accuracy but lacks large-scale observational evidence.

**Objective:**

To evaluate whether use of AI-assisted assessment for diagnosing clinically significant prostate cancer (csPCa) on MRI is superior to unassisted readings.

**Design, Setting, and Participants:**

This diagnostic study was conducted between March and July 2024 to compare unassisted and AI-assisted diagnostic performance using the AI system developed within the international Prostate Imaging-Cancer AI (PI-CAI) Consortium. The study involved 61 readers (34 experts and 27 nonexperts) from 53 centers across 17 countries. Readers assessed prostate magnetic resonance images both with and without AI assistance, providing Prostate Imaging Reporting and Data System (PI-RADS) annotations from 3 to 5 (higher PI-RADS indicated a higher likelihood of csPCa) and patient-level suspicion scores ranging from 0 to 100 (higher scores indicated a greater likelihood of harboring csPCa). Biparametric prostate MRI examinations were included for 780 men from the PI-CAI study who were included in the newly-conducted observer study. All men within the PI-CAI study had suspicion of harboring prostate cancer, sufficient diagnostic image quality, and no prior clinically significant cancer findings. Disease presence was defined by histopathology, and absence was determined by 3 or more years of follow-up. The AI system was recalibrated using 420 Dutch examinations to generate lesion-detection maps, with AI scores ranging from 1 to 10, in which 10 indicates the highest likelihood of csPCa. The remaining 360 examinations, originating from 3 Dutch centers and 1 Norwegian center, were included in the observer study.

**Main Outcomes and Measures:**

The primary outcome was diagnosis of csPCa, evaluated using the area under the receiver operating characteristic curve and sensitivity and specificity at a PI-RADS threshold of 3 or more. The secondary outcomes included analysis at alternate operating points and reader expertise.

**Results:**

Among the 360 examinations of 360 men (median age, 65 years [IQR, 62-70 years]) who were included for testing, 122 (34%) harbored csPCa. AI assistance was associated with significantly improved performance, achieving a 3.3% increase in the area under the receiver operating characteristic curve (95% CI, 1.8%-4.9%; *P* < .001), from 0.882 (95% CI, 0.854-0.910) in unassisted assessments to 0.916 (95% CI, 0.893-0.938) with AI assistance. Sensitivity improved by 2.5% (95% CI, 1.1%-3.9%; *P* < .001), from 94.3% (95% CI, 91.9%-96.7%) to 96.8% (95% CI, 95.2%-98.5%), and specificity increased by 3.4% (95% CI, 0.8%-6.0%; *P* = .01), from 46.7% (95% CI, 39.4%-54.0%) to 50.1% (95% CI, 42.5%-57.7%), at a PI-RADS score of 3 or more. Secondary analyses demonstrated similar performance improvements across alternate operating points and a greater benefit of AI assistance for nonexpert readers.

**Conclusions and Relevance:**

The findings of this diagnostic study of patients suspected of harboring prostate cancer suggest that AI assistance was associated with improved radiologic diagnosis of clinically significant disease. Further research is required to investigate the generalization of outcomes and effects on workflow improvement within prospective settings.

## Key Points

**Question:**

Is the use of a scientifically validated artificial intelligence (AI) system associated with improved diagnostic accuracy for detecting clinically significant prostate cancer (csPCa) on magnetic resonance imaging compared with unassisted readings?

**Findings:**

In this diagnostic study that included 61 readers and 360 prostate magnetic resonance imaging examinations among 360 male patients, AI assistance was associated with a statistically superior improvement in detecting csPCa, increasing the area under the receiver operating characteristic curve, sensitivity, and specificity compared with unassisted readings.

**Meaning:**

These findings suggest a potential added value of AI assistance during radiologic assessments to improve the diagnostic accuracy of csPCa.

## Teaser

This study assesses whether the use of a scientifically validated artificial intelligence (AI)-assisted system is associated with improved diagnostic accuracy for detecting clinically significant prostate cancer on magnetic resonance imaging (MRI) compared with an unassisted system.

## Introduction

Prostate cancer is the most commonly diagnosed cancer in men across 118 countries, accounting for more than 14% of cancers worldwide.^1^ Multiple studies have shown that magnetic resonance imaging (MRI)-targeted biopsies improve the detection of clinically significant prostate cancer (csPCa) while reducing unnecessary biopsies and the detection of insignificant prostate cancer, which has led to widespread adoption of prostate MRI in clinical practice.^2,3,4,5^ To standardize the reporting of prostate MRI, the Prostate Imaging Reporting and Data System (PI-RADS) was developed,^6^ providing readers with a 5-point Likert scale for csPCa diagnosis. Despite these advancements, diagnosis of csPCa using MRI remains challenging, showing considerable interreader variability and the need for high expertise.^7,8,9^ Meanwhile, the demand for prostate MRI is increasing worldwide.^10^

Multiple development efforts have demonstrated the potential of artificial intelligence (AI) in diagnosing csPCa on MRI.^11,12,13^ Integrating AI-assisted workflows may increase readers’ efficiency and improve variability while increasing or maintaining accurate csPCa diagnoses.^10,14^ Given the high stakes involved in clinical decision-making, concurrent AI workflows, in which readers can access AI-generated outcomes during prostate MRI assessments, have been explored. Various studies have shown that AI can potentially improve diagnostic performance, reduce interreader variability, and specifically boost accurate diagnoses for readers with less expertise.^15,16,17,18,19^ Most of these studies have, however, been limited by small datasets and reader cohorts. Additionally, the quality of AI algorithms is often not assessed through large-scale confirmatory studies, resulting in limited evidence of their efficacy.^20^

The Prostate Imaging-Cancer AI (PI-CAI) challenge is an international confirmatory study, in which a prostate AI system, designed for detection and diagnosis of csPCa, was developed and demonstrated to significantly outperform a large pool of readers in a robust, large-scale design.^21^ Building on this foundation, we conducted an international observer study and hypothesized that the assistance of the high-performing AI system would lead to significantly improved csPCa diagnosis compared with reader assessments without AI support. As secondary objectives, we evaluated whether AI assistance would provide greater diagnostic benefits to nonexpert readers compared with experts and analyzed diagnostic performance across different reader operating points.

## Methods

This diagnostic multireader, multicase observer study was conducted between March and July 2024 and incorporated retrospectively collected prostate MRI examinations and the AI system that was curated and developed within the international PI-CAI Consortium using 10 207 MRI examinations.^21^ Review boards at each contributing center approved the retrospective, anonymous reuse of image data and waived the requirement to obtain informed consent owing to the retrospective study design. The Standards for Reporting of Diagnostic Accuracy (STARD) reporting guideline was followed.

### Study Population

For this study, prostate MRI examinations from the PI-CAI cohort, acquired between 2015 and 2021 and originating from 4 European centers (Radboud University Medical Center [RUMC]; Ziekenhuisgroep Twente [ZGT]; Prostaat Centrum Noord-Nederland [PCNN]; and St Olavs Hospital, Trondheim University Hospital [STOH]), were included (refer to the CONSORT diagram in eFigure 1 in Supplement 1). Men with suspicion of prostate cancer due to elevated prostate-specific antigen (PSA) levels of 3 ng/mL or more (to convert to micrograms per liter, multiply by 1.0) and/or abnormal digital rectal examination findings, who subsequently underwent a prostate MRI examination, were included. Examinations with prior csPCa findings, prostate treatment, or severe imaging artifacts in the prostatic region were excluded (refer to Twilt et al^22^ for image quality examples).^21^ Imaging included biparametric prostate MRI examinations consisting of T2-weighted imaging in 3 planes, axial diffusion-weighted imaging with high *b* value images (*b* ≥ 1000 seconds/mm^2^), and apparent diffusion coefficient maps (details provided in Saha et al^21^). All imaging was acquired using 1.5T and 3T MRI systems from 2 vendors (Siemens Healthineers, Erlangen, Germany; Philips Medical Systems, Eindhoven, Netherlands). Patients with a positive MRI examination underwent a biopsy, while those with a negative MRI either did not undergo biopsy or received a systematic biopsy. Gleason grade group 2 or more, with Gleason scores of 3 and 4 or more, was used to define csPCa, whereas clinically insignificant prostate cancer was defined as Gleason grade 1.^23^ The presence of csPCa was determined by histopathology obtained using systematic and/or MRI-targeted biopsies. In cases in which patients underwent radical prostatectomy, whole-mount specimens were used as a reference. Absence of csPCa was confirmed with at least 3 years of follow-up. (For additional details on the curation of this study cohort, refer to Saha et al.^21^)

A total of 780 patients from the PI-CAI cohort were included in the newly-conducted observer study. All men within the PI-CAI study had suspicion of harboring prostate cancer, sufficient diagnostic image quality, and no prior clinically significant cancer findings and were included to establish a calibration cohort and a test cohort (eFigure 1 in Supplement 1). The calibration cohort comprised 420 examinations from RUMC, ZGT, and PCNN. This cohort included all 100 examinations from the hidden tuning cohort and 320 from the observer study conducted in the PI-CAI study, excluding examinations from STOH.^21^ The test cohort included 360 examinations from RUMC, ZGT, PCNN, and STOH, the latter serving as an unseen external center. Examinations in the test dataset were randomly sampled from the PI-CAI testing cohort, excluding examinations previously included in the observer study.^21^

### AI System

The AI system, developed and evaluated within the PI-CAI study, was used as a concurrent tool in this study.^21^ To summarize, AI architects within the PI-CAI study were trained and evaluated to diagnose and detect csPCa using a sequestered dataset of 10 207 biparametric MRI examinations from 4 European tertiary care centers. Each architecture produced a 3-dimensional volume with csPCa lesion detections with 0 to 100 likelihood scores of harboring csPCa (higher scores indicated a greater likelihood of csPCa), and an overall patient-level score for csPCa diagnosis ranging between 0 and 100 (higher scores indicated a greater likelihood of csPCa). The top 5 architectures from the PI-CAI study were combined into a single AI system, in which detection maps per algorithm were combined to create an average detection map (eAppendix 1 in Supplement 1). The lesion with the highest likelihood score was used as a patient-level score.

To enhance the interpretability of the AI system’s output for readers in this study and to achieve a uniform distribution of scores, the AI-generated detection map and patient-level scores were recalibrated to a scale of 1 to 10, in which 10 represented the highest likelihood of csPCa. This recalibration used the calibration dataset, ensuring that 10% of the calibration cohort was distributed evenly across each of the 10 AI score categories (eAppendix 1, eFigure 2, and eTables 1 and 2 in Supplement 1). Since the calibration cohort resembled characteristics of the test cohort, it was anticipated that approximately 10% of the test cohort would also fall into each score category.

### Observer Study

An observer study was conducted on the Grand Challenge platform from March 18, 2024, to July 12, 2024.^24^ During this time frame, 61 readers (53 centers from 17 countries) evaluated all examinations from the test cohort both with and without AI assistance. The readers (with a median of 5 years [IQR, 3-8 years] of experience in reading prostate MRI) were familiar with the PI-RADS, version 2.1, of which 43 (70%) practiced in clinical routine and 18 (30%) were in residency. Using self-reporting, 34 readers (56%) were categorized as experts (>1000 cases read in total and >200 cases per year), while the remaining 27 readers (44%) were categorized as nonexperts, following 2020 consensus statements from the European Society of Urogenital Radiology and the European Association of Urology (eFigure 3 and eTable 3 in Supplement 1).^25^ Before the start of the study, readers were provided with a detailed guide outlining the study objectives, the workstation setup, and the AI system, including its outputs and calibration process. Additionally, readers participated in a training session assessing 6 example examinations with and without AI assistance.

Readers and examinations were randomly divided into 6 substudies, stratified by center, csPCa prevalence, and reader experience. Each substudy contained 60 examinations, with 8 to 11 of the 61 total readers assigned to each split. The observer study used a crossover design, featuring 2 reading sessions separated by a 4-week washout period (eFigure 4 in Supplement 1). In the first phase, readers assessed 50% of their assigned examinations without AI assistance and the remaining 50% with AI assistance, organized into 4 alternating reading blocks. During assessments without AI assistance, readers had access to the full biparametric MRI protocol and were provided metadata associated with the patient (age, PSA level, prostate volume, and PSA density) and examination (MRI vendor and high *b* value). For AI-assisted assessments, readers were additionally provided with the AI system’s outputs, which included a lesion detection map displayed in a separate image port, overlaid on the T2 axial sequence, along with an overall patient-level score ranging from 1 to 10 (eFigure 5 in Supplement 1). For each examination, readers were asked to annotate and score lesions using the PI-RADS categories 3 to 5 and to provide an overall 0 to 100 patient-level score for csPCa diagnosis. Following the washout period, readers reassessed all examinations with the reading condition switched (ie, assessments that were previously performed without AI assistance were now done with AI assistance and vice versa), with the order of examinations reshuffled to minimize recall bias.

### Statistical Analysis

The primary objective was to assess whether AI-assisted csPCa diagnosis was superior to unassisted diagnosis at the patient level. This comparison was primarily evaluated using the area under the receiver operating characteristic curve (AUROC), along with sensitivity and specificity at a PI-RADS score of 3 or more. An a priori power analysis was conducted to determine the necessary number of readers and examinations to achieve a minimum of 80% power for this superiority test (eAppendix 2 and eTable 4 in Supplement 1).

ROCs and AUROCs were based on the patient-level suspicion scores provided during assessments. The highest PI-RADS score assigned by a reader was used as a patient-level score and was binarized at a PI-RADS score of 3 or more. Multireader, multicase analysis of variance using the Obuchowski–Rockette^26^ method was used to calculate mean estimates and 95% CIs based on Wald tests for the 3 diagnostic end points. The superiority of AI-assisted diagnosis was evaluated at a 2-sided *P* < .05 significance threshold and adjusted with Holm–Bonferroni correction for the 3 end points. Statistical tests and reporting of *P* values were reserved for the primary outcomes^27^ and were prespecified in a statistical analysis plan (eAppendix 3 and eFigure 6 in Supplement 1), using package MRMCaov, version 0.3.0, in R, version 2022.12.0 (R Project for Statistical Computing).

The secondary objectives were exploratory. The number of insignificant prostate cancer diagnoses was reported. To assess the association of expertise level with AI assistance outcomes, a subgroup analysis was performed categorizing expert and nonexpert readers. Diagnostic performance was evaluated at 2 additional reader operating points: (1) a PI-RADS score of 4 or more and a PI-RADS score of 3 with an elevated PSA density (≥0.15 ng/mL) and (2) a PI-RADS score of 4 or more only.^28^ The agreement between AI-assisted and unassisted assessments was summarized quantitively.

## Results

### Patient Population

A total of 360 MRI examinations were assessed in the observer study involving 360 male patients with a median age of 65 years (IQR, 62-70 years) and a median PSA level of 7.0 ng/mL (IQR, 5.2-10.0 ng/mL). Among these examinations, 122 of the 360 patients (34%) were diagnosed with csPCa. Patient characteristics for the testing cohort are provided in the Table. Detailed characteristics of the 6 split-plot designs are provided in eTable 5 in Supplement 1, while characteristics of the calibration cohort are available in eTable 6 in Supplement 1.

**Table.** Testing Cohort Characteristics^a^

Characteristic	Total (N = 360)	RUMC (n = 113)	ZGT (n = 97)	PCNN (n = 76)	STOH (n = 74)
Patient age, median (IQR), y	65 (62-70)	67 (61-71)	64 (62-68)	68 (63-72)	64 (58-68)
Patient PSA level, median (IQR), ng/mL	7.0 (5.2-10.0)	6.7 (5.2-10.0)	6.5 (5.1-8.8)	8.6 (6.0-11.2)	6.9 (5.0-10.9)
Patient prostate volume, median (IQR), mL	55.0 (40.0-77.0)	68.0 (48.0-98.0)	55.0 (40.0-72.0)	48.0 (36.0-61.0)	48.0 (37.0-66.0)
Patient PSAd, median (IQR), ng/mL^2^	0.13 (0.09-0.21)	0.10 (0.07-0.16)	0.13 (0.09-0.20)	0.18 (0.11-0.25)	0.13 (0.09-0.22)
Patient csPCa status					
Without	238 (66)	84 (74)	65 (67)	44 (58)	45 (61)
With	122 (34)	29 (26)	32 (33)	32 (42)	29 (39)
MRI vendor					
Siemens Healthineers	301 (84)	113 (100)	97 (100)	17 (22)	74 (100)
Philips Medical Systems	59 (16)	0	0	59 (78)	0
Field strength, T					
1.5	14 (4)	0	0	14 (18)	0
3	346 (96)	113 (100)	97 (100)	62 (82)	74 (100)
Ground truth verification					
No Bx, follow-up^b^	61 (17)	52 (46)	0	0	9 (12)
Sys Bx	126 (35)	12 (11)	44 (45)	32 (42)	38 (51)
MRGBx	47 (13)	10 (9)	0	35 (46)	2 (3)
Sys Bx and MRGBx	78 (22)	31 (27)	35 (36)	0	12 (16)
RP	48 (13)	8 (7)	18 (19)	9 (12)	13 (18)
Gleason grade					
0	186 (52)	77 (68)	43 (44)	27 (36)	39 (53)
1	52 (14)	7 (6)	22 (23)	17 (22)	6 (8)
2	57 (16)	14 (12)	17 (18)	18 (24)	8 (11)
3	31 (9)	7 (6)	5 (5)	8 (11)	11 (15)
4	10 (3)	1 (1)	1 (1)	4 (5)	4 (5)
5	24 (7)	7 (6)	9 (9)	2 (3)	6 (8)
PI-RADS score from original report^c^					
1-2	171 (48)	65 (58)	45 (46)	24 (32)	37 (50)
3	27 (8)	6 (5)	7 (7)	9 (12)	5 (7)
4	80 (22)	24 (21)	18 (19)	29 (38)	9 (12)
5	82 (23)	18 (16)	27 (28)	14 (18)	23 (31)
AI score					
1	34 (9)	9 (8)	18 (19)	3 (4)	4 (5)
2	50 (14)	24 (21)	11 (11)	5 (7)	10 (14)
3	33 (9)	14 (12)	8 (8)	4 (5)	7 (9)
4	31 (9)	6 (5)	7 (7)	6 (8)	12 (16)
5	49 (14)	17 (15)	11 (11)	12 (16)	9 (12)
6	26 (7)	10 (9)	5 (5)	7 (9)	4 (5)
7	38 (11)	10 (9)	10 (10)	13 (17)	5 (7)
8	27 (8)	6 (5)	8 (8)	11 (14)	2 (3)
9	30 (8)	7 (6)	9 (9)	10 (13)	4 (5)
10	42 (12)	10 (9)	10 (10)	5 (7)	17 (23)

Abbreviations: AI, artificial intelligence; Bx, biopsy; csPCa, clinically significant prostate cancer (Gleason grade ≥2); MRGBx, magnetic resonance imaging (MRI)-guided Bx; PCNN, Prostaat Centrum Noord-Nederland, Netherlands; PI-RADS, Prostate Imaging Reporting and Data System; PSA, prostate-specific antigen; PSAd, PSA density; RP, radical prostatectomy; RUMC, Radboud University Medical Center, Netherlands; STOH, St Olavs Hospital, Trondheim University Hospital, Norway; Sys Bx, systematic ultrasound-guided Bx; ZGT, Ziekenhuisgroep Twente, Netherlands.

SI conversion factor: To convert PSA level to micrograms per liter, multiply by 1.0.

^a^ Data are presented as No. (%) of patients unless otherwise indicated.

^b^ Follow-up period of at least 3 years.

^c^ Defined as the highest score found on a per-patient level as assigned in the original radiology report from routine clinical practice.

### Diagnostic Performances

**Figure 1.**
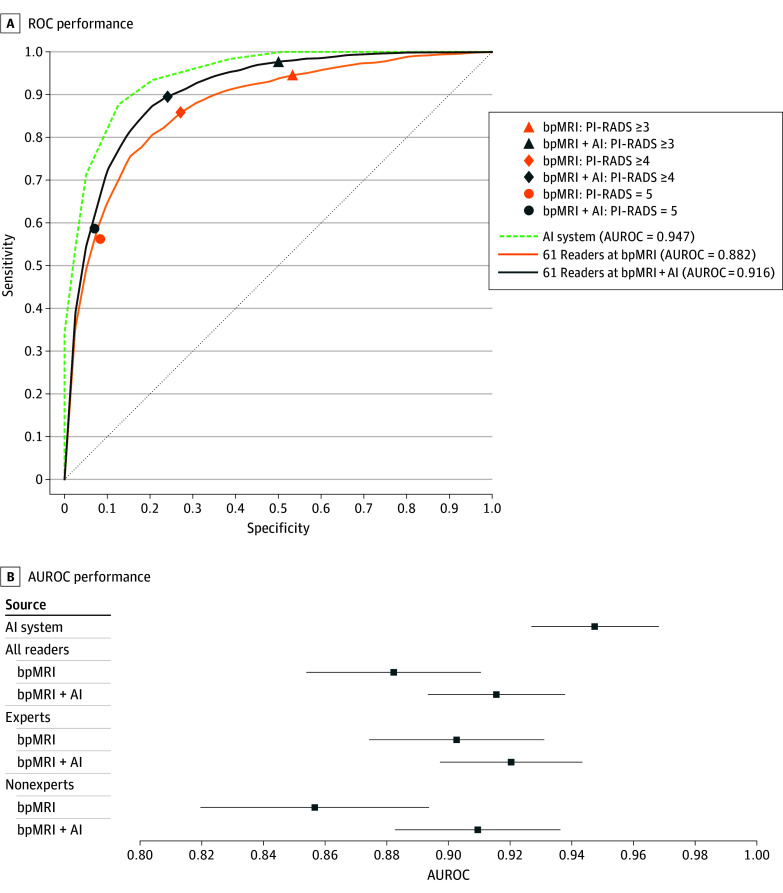
The Area Under the Receiver Operating Characteristic Curve (AUROC) Diagnostic Performance at Biparametric Magnetic Resonance Imaging (bpMRI) and at bpMRI With Artificial Intelligence Assistance (bpMRI + AI) A, ROCs of the performances of the AI system and the pool of 61 readers at bpMRI and bpMRI + AI. The diagonal dashed line indicates a random classifier. B, AUROC performance for the stand-alone AI system, all readers (N = 61), and subgroups considering expert (n = 34) and nonexpert (n = 27) readers for assessment made at bpMRI and bpMRI + AI at a Prostate Imaging Reporting and Data System (PI-RADS) score of 3 or more. Expert readers are readers with more than 1000 cases read in total and more than 200 cases per year, following 2020 consensus statements from the European Society of Urogenital Radiology and the European Association of Urology. Markers indicate mean AUROC; error bars, 95% CIs.

**Figure 2.**
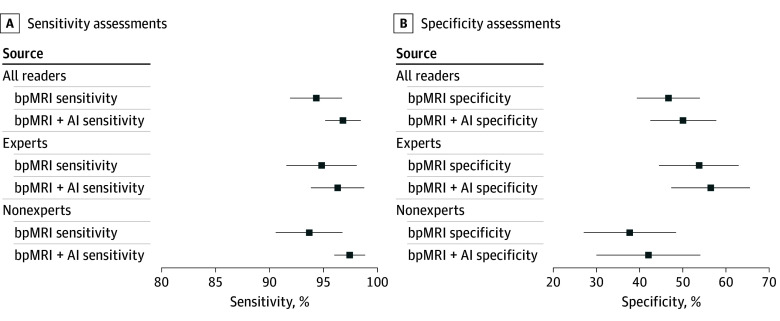
Sensitivity and Specificity at Biparametric Magnetic Resonance Imaging (bpMRI) Assessments and at bpMRI Assessments With Artificial Intelligence Assistance (bpMRI + AI) Sensitivities (A) and specificities (B) for all 61 readers and subgroups considering experts (n = 34) and nonexperts (n = 27) at a Prostate Imaging Reporting and Data System operating point of 3 or more. Expert readers are readers with more than 1000 cases read in total and more than 200 cases per year, following 2020 consensus statements from the European Society of Urogenital Radiology and the European Association of Urology. Markers indicate mean percentages; error bars, 95% CIs.

Figure 1 and Figure 2 highlight the diagnostic performances of the AI system and readers across all primary end points. The AUROC of readers when assessing biparametric MRI with AI assistance was 0.916 (95% CI, 0.893-0.938) compared with 0.882 (95% CI, 0.854-0.910) for unassisted assessments, demonstrating a superior improvement of 3.3% (95% CI, 1.8%-4.9%; *P* < .001). Notably, the stand-alone AI system had a higher AUROC (0.947 [95% CI, 0.927-0.968]) than readers at both reading conditions.

At the PI-RADS operating point of 3 or more, AI-assisted assessments demonstrated a sensitivity of 96.8% (95% CI, 95.2%-98.5%) compared with 94.3% (95% CI, 91.9%-96.7%) for unassisted assessments, representing a significant improvement of 2.5% (95% CI, 1.1%-3.9%; *P* < .001) and resulting in 3 additional true positive diagnoses (eFigure 7 in Supplement 1). Similarly, specificity was significantly higher with AI assistance, increasing by 3.4% (95% CI, 0.8%-6.0%; *P* = .01) to 50.1% (95% CI, 42.5%-57.7%) compared with 46.7% (95% CI, 39.4%-54.0%) in unassisted assessments. The number of false positive diagnoses was reduced by 10 (eTable 7 in Supplement 1). Under both reading conditions, the mean number of insignificant prostate cancer diagnoses was similar (35 [IQR, 24-42] at biparametric MRI without AI assistance and 35 [IQR, 24-48] at biparametric MRI with AI assistance). The differences in diagnostic performances of all individual readers are provided in eFigure 8 in Supplement 1.

Subset analyses, as illustrated in Figure 1 and Figure 2, highlight the outcome of AI assistance based on reader expertise. Nonexpert readers derived greater benefit from AI assistance than experts, with a difference in AUROC of 0.053 (95% CI, 0.028-0.078) for nonexperts vs 0.018 (95% CI, 0.001-0.034) for experts. Sensitivity improvements were 3.7% (95% CI, 1.3%-6.2%) for nonexperts vs 1.5% (95% CI, 0.3%-3.3%) for experts, while specificity gains were 4.3% (95% CI, 0.4%-9.0%) for nonexperts vs 2.8% (95% CI, 0%-5.6%) for experts. AI assistance provided an additional 1% reduction in false positive and a 2% increase in true negative diagnoses compared with experts (eFigure 7 in Supplement 1). Across the 2 alternate reader operating points, an overall improvement in sensitivity and specificity was observed for evaluations with AI assistance (eTable 7 in Supplement 1).

### Quantitative Comparison Between Unassisted and AI-Assisted Assessments

**Figure 3.**
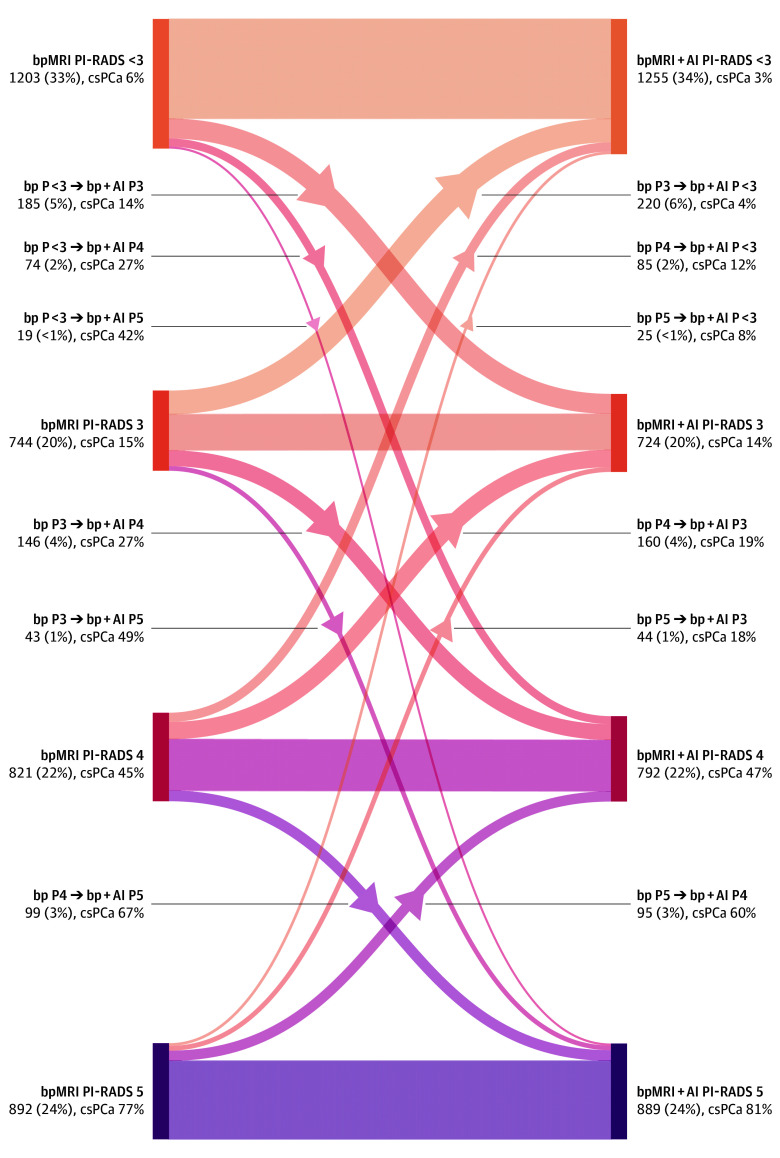
Diagram of Patient-Level Prostate Imaging Reporting and Data System (PI-RADS) Scores From Unassisted Biparametric Magnetic Resonance Imaging (bpMRI; Left) and Artificial Intelligence (AI)-Assisted bpMRI (bpMRI + AI; Right) Assessments in the Observer Study The diagram highlights interrater consistencies and changes (upgrades and downgrades) between the 2 configurations. Each scoring category and pair is presented with occurrence numbers, percentages, and clinically significant prostate cancer (csPCa) prevalence. Of all readings, 2465 of the 3660 total assessments (67%) remained unchanged between assessments, 278 (8%) involved reclassification from negative (PI-RADS [P] <3) to positive (PI-RADS [P] ≥3) MRI, and 330 (9%) involved reclassification from positive to negative MRI. The remaining 587 (16%) involved reclassification within the positive MRI group. The overall PI-RADS score distribution was similar across both reading configurations, while csPCa prevalence changed due to scoring updates.

In 1195 (33%) of the 3660 evaluations in the observer study, readers assigned different patient-level scores between the 2 reading conditions (Figure 3). Among these differences, 566 (15%) were upgrades, and 629 (17%) were downgrades under AI-assisted reading. Specifically, 278 (8%) involved redesignation from an initial negative MRI (PI-RADS score <3) to a positive MRI (PI-RADS score ≥3), while 330 (9%) concerned reclassifications from a positive MRI to a negative MRI. In 246 of 360 examinations (68%), at least 1 reader who was assigned to a given examination reclassified their diagnosis, with a median of 1 reclassification per examination (range 0-7; IQR, 0-3).

Despite these updates, the overall distribution of PI-RADS scores remained similar between reading conditions. However, AI-assisted assessments demonstrated a 3% decrease in the prevalence of csPCa for a PI-RADS score of 1 to 2, lowering the prevalence of csPCa from 6% to 3%. For a PI-RADS score of 3, csPCa prevalence decreased by 1%, while it increased by 2% for a PI-RADS score of 4 and 4% for a PI-RADS score of 5. Examples of assessments on various examinations are shown in eFigures 9-14 in Supplement 1.

**Figure 4.**
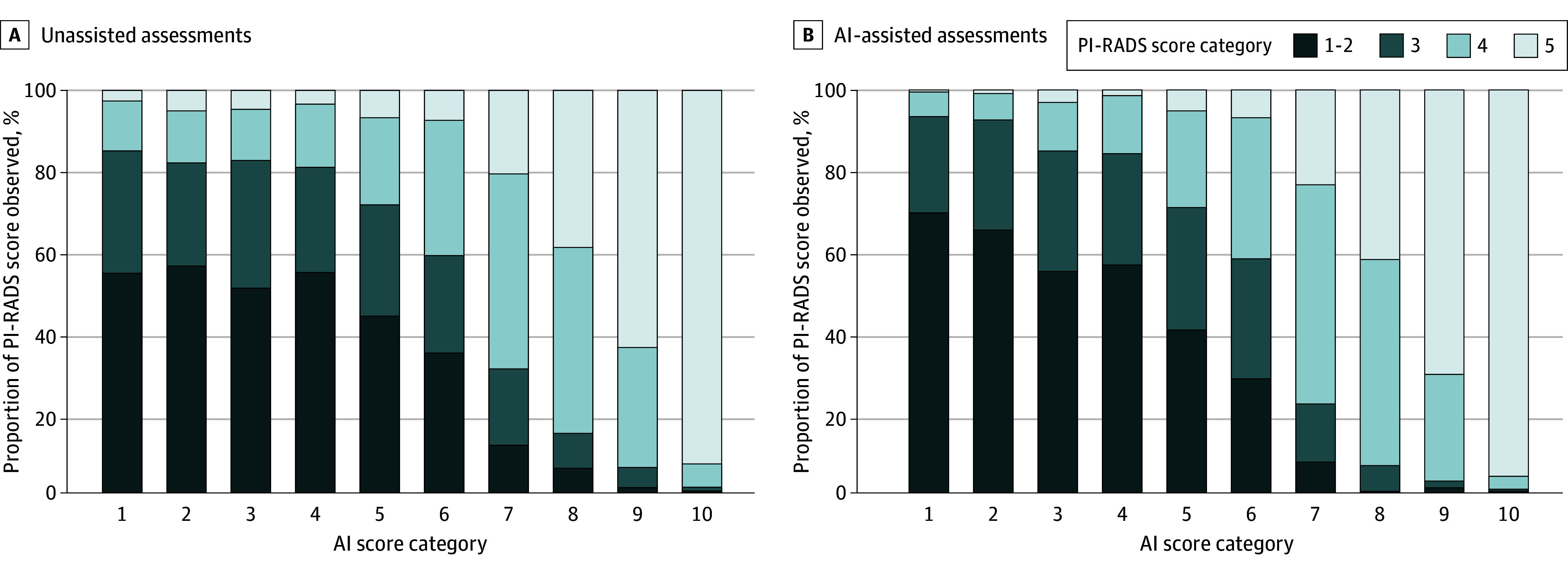
Proportion of Prostate Imaging Reporting and Data System (PI-RADS) Scores Observed for Unassisted and Artificial Intelligence (AI)-Assisted Assessments by AI Scores of Examinations Among the 3660 total assessments, AI assistance (right) compared with unassisted assessment (left) was associated with increases in the PI-RADS score of 1 to 2 in lower AI score categories and with decreases in higher AI score categories. Similarly, the proportion of PI-RADS scores of 4 to 5 increased in high AI score categories with AI assistance.

To further explore the association of AI assistance with reader assessments, Figure 4 presents the proportion of PI-RADS scores across AI score categories for both unassisted and AI-assisted assessments. For examinations with AI score categories below 5, AI assistance was associated with a higher proportion of a PI-RADS score of 1 to 2. At higher AI scores,^7,8,9,10^ AI-assisted assessments showed a greater proportion of PI-RADS scores of 4 and 5 compared with unassisted assessments. Notably, PI-RADS scores of 3 increased for AI scores of 4 (27% vs 26%), 5 (30% vs 27%), and 6 (29% vs 24%), indicating higher equivocal diagnoses for these categories. eFigure 15 in Supplement 1 presents the proportion of PI-RADS scores across AI scores for expertise subgroups.

## Discussion

In this diagnostic study, a publicly developed and benchmarked AI system was implemented as a concurrent tool to assist readers in evaluating prostate MRI, aiming to assess whether there was a degree of improvement for csPCa diagnosis. Through a large-scale, international observer study involving 61 readers, we demonstrated that AI assistance was associated with a significant improvement in csPCa diagnosis, as shown by a superior AUROC (0.916 vs 0.882; *P* < .001), as well as significantly improved sensitivity (96.8% vs 94.3%; *P* < .001) and specificity (50.1% vs 46.7%; *P* = .01) at a PI-RADS operating point of 3 or more. Stratifying readers by experience showed that nonexperts gained greater benefits from AI support than experts did. Interestingly, the overall distribution of PI-RADS scores remained largely unchanged; however, AI exhibited both positive and negative associations with reader assessments.

Consistent with prior research, our findings support the role of AI assistance associated with improved csPCa diagnosis.^15,16,17,18,19^ Our study strengthens this evidence by using multicenter data involving a large international cohort of readers and an AI system benchmarked through an international confirmatory study, demonstrating high diagnostic performance.^21^ The sensitivity and specificity at a PI-RADS score of 3 or more aligned with the pooled sensitivity of 96% (95% CI, 93%-98%) and specificity of 49% (95% CI, 29%-70%) reported by the systematic review and meta-analysis from Woo et al.^29^ However, comparisons should be interpreted cautiously due to population and methodologic differences. The diagnostic gains from AI support in our study were smaller than previous studies conducted on a smaller scale.^16,17,18,19,30^ Sun et al^15^ reported comparable sensitivity gains (88.3% to 93.9%) but greater specificity improvements (57.7% to 71.7%) in a study with 480 cases and 16 readers. Differences in AI benefits may originate from the inclusion of a multicenter dataset and a large, diverse group of international readers with varying expertise.

With AI assistance, PI-RADS scores were updated in 33% of assessments, including 17% involving reclassification between positive and negative MRI results, which likely altered the biopsy decision for these patients. AI assistance was associated with improved csPCa detection in PI-RADS categories 4 and 5 and reduced detection in the PI-RADS category 1 to 2 from 6% to 3%. The latter prevalence is below the population risk and potentially allowed for increased confidence for safe discharge from primary diagnostic settings and reduced unnecessary biopsies with AI assistance.^28^ Lower AI scores were associated with increased PI-RADS scores of less than 3, while higher AI scores were associated with increased PI-RADS scores of 4 and 5, indicating a greater reliance on AI in these categories. In contrast, intermediate AI scores were associated with increases in both PI-RADS category 3 and PI-RADS category 4 assessments, reflecting elevated reader suspicion of csPCa.

Furthermore, the overall distribution of PI-RADS scores remained consistent across reading settings, and AI assistance was not associated with reducing equivocal diagnoses, contrasting previous findings.^16,19^ Those studies incorporated AI systems with scoring scales resembling PI-RADS, which may have facilitated the interpretation of AI outcomes by aligning them more closely with familiar diagnostic categories. A potential limitation of these approaches is that these scores do not necessarily adhere to the ordinal scale and the standardized categorization rules defined in PI-RADS, version 2.1,^6^ and may create confusion regarding the calibration and definition of AI scores. The proportion of PI-RADS category 3 in our study was comparable with that reported in a recent prospective study^31^ but 3% higher than the prevalence reported in the systematic review and meta-analysis by Maggi et al.^32^ This increase may be attributed to the larger proportion of less-experienced readers,^3^ variability in image quality, and assessments conducted outside familiar reading environments. The persistent proportion of equivocal diagnoses may additionally suggest a greater reluctance to miss a cancer diagnosis than to reduce unnecessary biopsies.

Prior research suggests that AI specifically enhances the performance of nonexpert readers.^16,17,33^ Consistent with these findings, our study suggests that nonexperts experienced a greater performance boost from AI assistance compared with experts, highlighting the potential of AI to reduce performance differences between experts and nonexperts. Nonexperts with AI support achieved higher AUROC scores than experts without AI, and their sensitivity surpassed that of experts in both unassisted and AI-assisted settings. While AI assistance was also associated with improved specificity for nonexperts, it did not reach the level of experts’ unassisted specificity, and performance differences among nonexperts remained considerable. These findings suggest that while nonexperts may adhere to AI recommendations,^34,35^ they often retain their incorrect detections associated with an aversion to missing cancer diagnoses.

Although AI assistance was associated with improved overall reader performance, AI as a stand-alone system outperformed both unassisted and AI-assisted readers, highlighting the potential for more optimized integration of AI in the diagnostic workflow. From one perspective, readers could have potentially gained more benefits from AI by an increased training period, in which they would optimize their understanding of risk scores and their integration in decision-making. Alternatively, diagnostic accuracy can be potentially boosted by the integration of independent AI. However, the widespread adoption of such stand-alone AI systems is currently hindered by a lack of prospective evidence, ethical concerns, and regulatory challenges.^12,14,36^ To bridge this gap, these systems could be introduced to autonomously diagnose specific population subsets, in which AI demonstrates high confidence, supplemented by reader assessments and multidisciplinary oversight prior to biopsy decisions. Such integrations hold the potential to improve both diagnostic performance and workload efficiency.

### Limitations

Multiple limitations of this study are acknowledged. First, the data included were retrospectively curated within the scope of PI-CAI,^21^ resulting in a mix of consecutive and sampled cohorts, mostly originating from a single MRI manufacturer. Second, the current study specifically investigated the use of concurrent AI within a cohort in which the AI system had previously demonstrated a strong diagnostic performance, without assessing its generalizability to different cohorts. The objective of this study was to implement a validated, high-performing AI system to evaluate its association with csPCa diagnosis within an assistive setting.^36^ Performance evaluation on new external data lies beyond the scope of this study, yet it is essential for broader clinical implementation. Future research should focus on identifying failure cases and those outside the training distribution, particularly across external cohorts with varying disease prevalence, image quality, and other clinical and demographic factors. Third, readers in this study assessed examinations through a controlled online reading workstation, which may have differed considerably from their native environments and may have impacted diagnostic performance. Fourth, this study did not assess workflow efficiency or the clinical applicability of performance improvements. Evaluating these aspects requires deployment in real or simulated clinical settings, considering the full spectrum of the MRI diagnostic pathway.^37^ Last, not all patients with negative MRI underwent histopathologic confirmation, and the decision to biopsy was based on multiparametric MRI readings from clinical routine rather than outcomes from biparametric MRI assessments or AI predictions.

## Conclusions

The findings of this diagnostic study suggest the potential of AI assistance in improving csPCa diagnosis when compared with unassisted assessments of biparametric MRI, with statistically significant improvements observed across AUROC, sensitivity, and specificity at a PI-RADS score of 3 or more. Notably, nonexpert readers demonstrated higher benefits from AI assistance compared with expert readers. There is a need for continued exploration of human-AI interactions, along with the prospective deployment of AI in a clinical setting to assess the generalizability of our findings and to evaluate its impact on workflow efficiency.

## Notes

Supplement 1. **eFigure 1.** CONSORT Diagram **eAppendix 1.** Overview of AI System and Calibration **eFigure 2.** Effects of Recalibration **eTable 1.** Performance Metrics for the AI System in the Calibration Cohort **eTable 2.** Performance of Readers Within the PI-CAI Reader Study for Comparison **eFigure 3.** Reader Characteristics **eTable 3.** Reader Characteristics per Split-Plot in the Observer Study **eFigure 4.** Schematic Representation of the Reader Study Design **eFigure 5.** Reader Study Design and Interface **eAppendix 2.** Power Analysis **eTable 4.** Standard Errors and Power Estimates **eAppendix 3.** Statistical Analysis Plan **eFigure 6.** Objectives for Primary Outcomes **eTable 5.** Split-Plot Data and Reader Characteristics **eTable 6.** Calibration Cohort Characteristics **eFigure 7.** Proportion of Diagnoses Across Unassisted and AI-Assisted Assessments Including Expertise Subgroups **eTable 7.** Performance Metrics Across Alternate Operating Points **eFigure 8.** Individual Performance Differences of Readers **eFigure 9.** Example of AI-Assisted Upgrading in the Assessment of a Patient With Clinically Significant Prostate Cancer **eFigure 10.** Example of AI-Assisted Downgrading in the Assessment of a Patient With Clinically Significant Prostate Cancer **eFigure 11.** Example of AI-Assisted Downgrading in the Assessment of a Patient Without Clinically Significant Prostate Cancer **eFigure 12.** Example of AI-Assisted Downgrading in the Assessment of a Patient Without Clinically Significant Prostate Cancer **eFigure 13.** Example of AI-Assisted Upgrading in the Assessment of a Patient Without Clinically Significant Prostate Cancer **eFigure 14.** Example of AI-Assisted Upgrading in the Assessment of a Patient Without Clinically Significant Prostate Cancer **eFigure 15.** Proportion of PI-RADS Scores Across Unassisted and AI-Assisted Assessments for Expertise Subgroups
eReferences

Supplement 2.
Nonauthor Collaborators

Supplement 3.
Data Sharing Statement
