# Supplementary material for: AI-Assisted vs Unassisted Identification of Prostate Cancer in Magnetic Resonance Images
Source: JAMA Netw Open. 2025 Jun 13;8(6):e2515672. doi: 10.1001/jamanetworkopen.2025.15672 (PMC12166490; doi:10.1001/jamanetworkopen.2025.15672)
Supplement: Supplement 1. — eFigure 1. CONSORT Diagram eAppendix 1. Overview of AI System and Calibration eFigure 2. Effects of Recalibration eTable 1. Performance Metrics for the AI System in the Calibration Cohort eTable 2. Performance of Readers Within the PI-CAI Reader Study for Comparison eFigure 3. Reader Characteristics eTable 3. Reader Characteristics per Split-Plot in the Observer Study eFigure 4. Schematic Representation of the Reader Study Design eFigure 5. Reader Study Design and Interface eAppendix 2. Power Analysis eTable 4. Standard Errors and Power Estimates eAppendix 3. Statistical Analysis Plan eFigure 6. Objectives for Primary Outcomes eTable 5. Split-Plot Data and Reader Characteristics eTable 6. Calibration Cohort Characteristics eFigure 7. Proportion of Diagnoses Across Unassisted and AI-Assisted Assessments Including Expertise Subgroups eTable 7. Performance Metrics Across Alternate Operating Points eFigure 8. Individual Performance Differences of Readers eFigure 9. Example of AI-Assisted Upgrading in the Assessment of a Patient With Clinically Significant Prostate Cancer eFigure 10. Example of AI-Assisted Downgrading in the Assessment of a Patient With Clinically Significant Prostate Cancer eFigure 11. Example of AI-Assisted Downgrading in the Assessment of a Patient Without Clinically Significant Prostate Cancer eFigure 12. Example of AI-Assisted Downgrading in the Assessment of a Patient Without Clinically Significant Prostate Cancer eFigure 13. Example of AI-Assisted Upgrading in the Assessment of a Patient Without Clinically Significant Prostate Cancer eFigure 14. Example of AI-Assisted Upgrading in the Assessment of a Patient Without Clinically Significant Prostate Cancer eFigure 15. Proportion of PI-RADS Scores Across Unassisted and AI-Assisted Assessments for Expertise Subgroups eReferences [file jamanetwopen-e2515672-s001.pdf]

Supplemental Online Content

Twilt JJ, Saha A, Bosma JS, et al.; on behalf of the PI-CA Consortium. AI-assisted vs unassisted identification of prostate cancer in magnetic resonance images. *JAMA Netw Open*. 2025;8(6):e2515672. doi:10.1001/jamanetworkopen.2025.15672

eFigure 1. CONSORT Diagram
eAppendix 1. Overview of AI System and Calibration
eFigure 2. Effects of Recalibration
eTable 1. Performance Metrics for the AI System in the Calibration Cohort
eTable 2. Performance of Readers Within the PI-CAI Reader Study for Comparison
eFigure 3. Reader Characteristics
eTable 3. Reader Characteristics per Split-Plot in the Observer Study
eFigure 4. Schematic Representation of the Reader Study Design
eFigure 5. Reader Study Design and Interface
eAppendix 2. Power Analysis
eTable 4. Standard Errors and Power Estimates
eAppendix 3. Statistical Analysis Plan
eFigure 6. Objectives for Primary Outcomes
eTable 5. Split-Plot Data and Reader Characteristics
eTable 6. Calibration Cohort Characteristics
eFigure 7. Proportion of Diagnoses Across Unassisted and AI-Assisted Assessments Including Expertise Subgroups
eTable 7. Performance Metrics Across Alternate Operating Points
eFigure 8. Individual Performance Differences of Readers
eFigure 9. Example of AI-Assisted Upgrading in the Assessment of a Patient With Clinically Significant Prostate Cancer
eFigure 10. Example of AI-Assisted Downgrading in the Assessment of a Patient With Clinically Significant Prostate Cancer
eFigure 11. Example of AI-Assisted Downgrading in the Assessment of a Patient Without Clinically Significant Prostate Cancer
eFigure 12. Example of AI-Assisted Downgrading in the Assessment of a Patient Without Clinically Significant Prostate Cancer
eFigure 13. Example of AI-Assisted Upgrading in the Assessment of a Patient Without Clinically Significant Prostate Cancer
eFigure 14. Example of AI-Assisted Upgrading in the Assessment of a Patient Without Clinically Significant Prostate Cancer
eFigure 15. Proportion of PI-RADS Scores Across Unassisted and AI-Assisted Assessments for Expertise Subgroups
eReferences

This supplemental material has been provided by the authors to give readers additional information about their work.

© 2025 Twilt JJ et al. *JAMA Network Open*.

eFigure 1. CONSORT Diagram

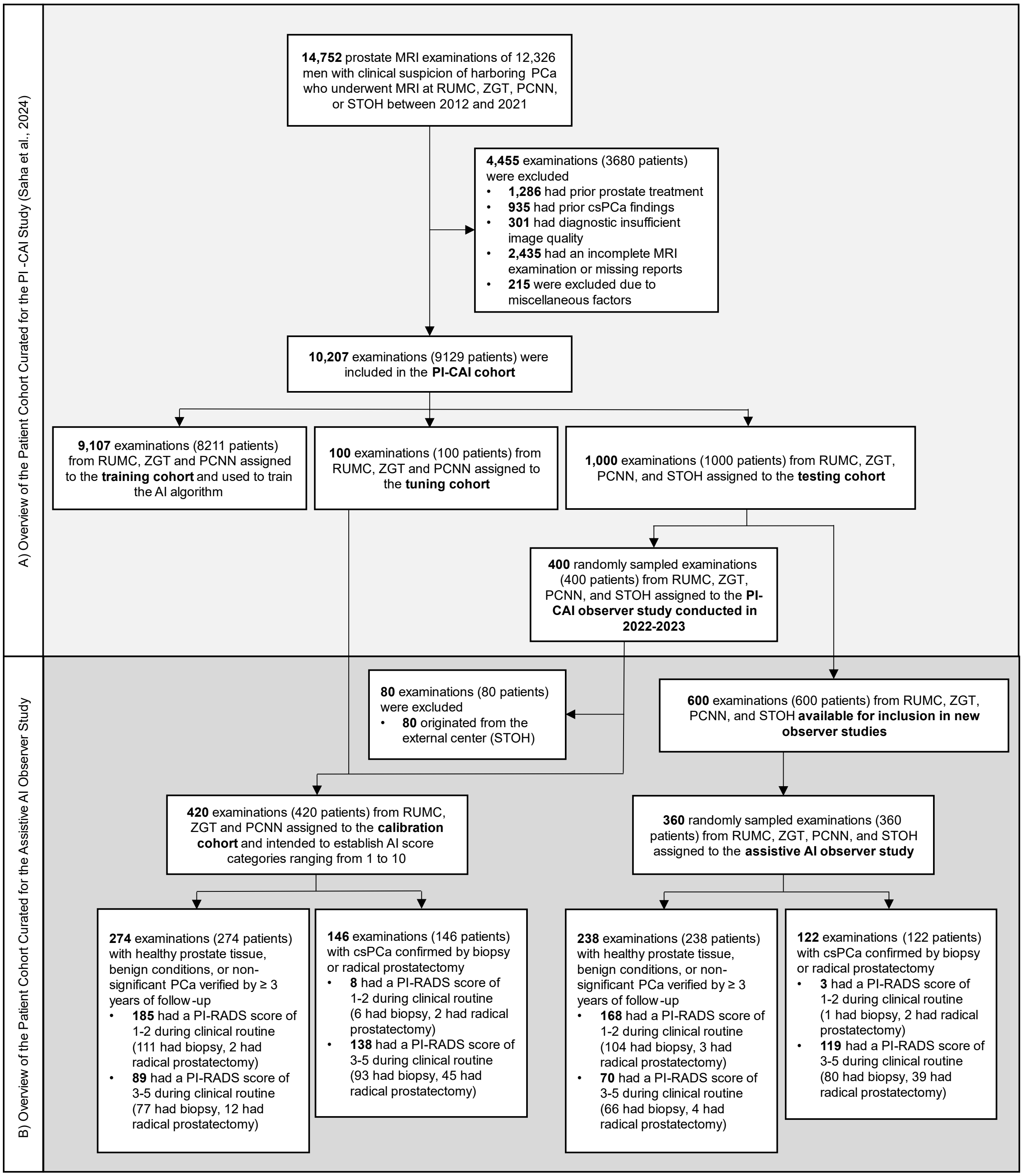

eFigure 1 – CONSORT diagram summarizing the patient inclusion for (A) the PI-CAI study [1] and (B) the newly-conducted assistive AI observer study. Patients with suspicion of harboring prostate cancer (PCa) who underwent prostate MRI originated from Radboud University Medical Center (RUMC), Ziekenhuisgroep Twente (ZGT), Prostaat Centrum Noord-Nederland (PCNN) and St. Olav's Hospital, Trondheim University Hospital (STOH). For calibration, patients were resampled from the PI-CAI tuning cohort and the prior conducted observer study, while the new AI-assisted observer study included patients from the PI-CAI testing cohort who had not been part of the previous observer study.

© 2025 Twilt JJ et al. *JAMA Network Open*.

eAppendix 1. Overview of AI System and Calibration

The AI system deployed in this study consisted of an equally weighted ensemble model from the top-five ranking AI models in the PI-CAI challenge developed by N. Debs et al. (Guerbet Research, France), Y. Yuan et al. (University of Sydney, Australia), H. Kan et al. (University of Science and Technology, China), C. Li et al. (Stanford University, United States) and A. Karagöz et al. (Istanbul Technical University, Turkey) [1-6]. These algorithms are publicly accessible through the PI-CAI challenge platform at https://pi-cai.grand-challenge.org/algorithms. All models were based on deep convolutional neural networks and employed biparametric axial-plane imaging sequences. Four of the five models incorporated a preprocessing step for prostate zonal segmentation to minimize false-positive lesion predictions. Additionally, two models integrated patient-specific clinical data (age, prostate-specific antigen [PSA] levels and prostate volume) into their architectures. Each model outputs a lesion detection map, where for each predicted lesion all voxels comprise a single floating point between 0-1.

The five individual detection maps were averaged to create an ensembled prediction map. Lesion candidates were then identified to construct a final ensembled detection map. The first lesion was identified as the voxel region with the highest level of suspicion. New candidates were identified iteratively until a maximum of five candidates were found, or until any candidates consisted of fewer than 10 voxels, or when the highest remaining suspicion dropped below 0.01 [7]. The highest suspicion score within the detection map served as the patient-level diagnosis score.

Following internal peer review, the AI model scores were recalibrated to enhance interpretability for radiologists. This adjustment was required, as deep learning ensemble models inherently lack transparency in their score generation, making uncalibrated outputs difficult to interpret. Utilizing the calibration cohort (n=420 examinations) the ensembled detection map and patient-level scores were recalibrated to a 1-10 scale, where a score of 10 indicates a high likelihood of clinically significant prostate cancer (csPCa). The calibration process evenly distributed 10% of the calibration cohort into each score category. A comparison between uncalibrated and calibrated AI scores in eFigure 2 highlights the effect of recalibration. eTable 2 presents performance metrics for the AI system in the calibration cohort, whereas eTable 3 presents the performance of readers within the PI-CAI reader study for comparison [1]. Readers were provided with these details before the start of the study to ensure a sufficient understanding of the AI system and its outputs.

eFigure 2. Effects of Recalibration

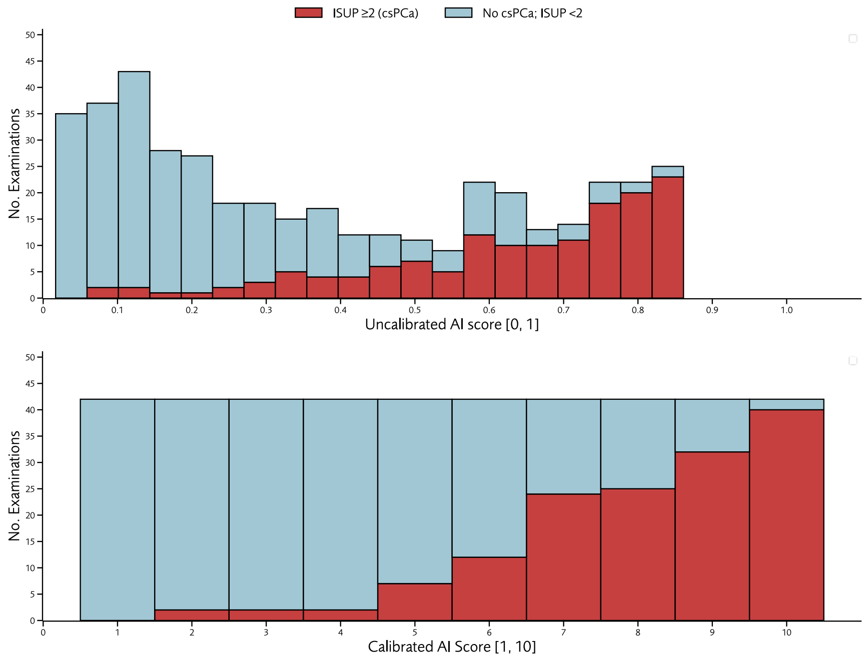

© 2025 Twilt JJ et al. *JAMA Network Open*.

eFigure 2 – Histogram illustrating the distribution of examinations within the calibration cohort (n=420) ordering examinations according to the uncalibrated AI score (range 0 to 1) (upper) and calibrated AI score (range 1 to 10) (bottom) and according to the presence of clinically significant prostate cancer (csPCa, Gleason Grade group 2/ISUP 2). The calibration ensured each AI score category contained 10% of the total calibration dataset. Readers were provided with the same table in the instruction manual.

eTable 1. Performance Metrics for the AI System in the Calibration Cohort

Prevalence of clinically significant prostate cancer (csPCa, Gleason Grade group 2) and sensitivity, specificity, positive predictive value (PPV) and negative predictive value (NPV) across AI score categories in the calibration dataset (n=420, csPCa prevalence of 35%). Prevalence is provided within the AI score group, whereas performance metrics are provided for AI score thresholds. Readers were provided with the same table in the instruction manual.

AI Score	csPCa Prevalence Group (n, [%])	Non Disease Prevalence Group (n, [%])	Sensitivity (%)	Specificity (%)	PPV (%)	NPV (%)
1	0	42 (100)	100	0	34.8	0
2	2 (5)	40 (95)	100	15.3	38.6	100
3	2 (5)	40 (95)	98.6	29.9	42.9	97.6
4	2 (5)	40 (95)	97.3	44.5	48.3	96.8
5	7 (17)	35 (83)	95.9	59.1	55.6	96.4
6	12 (29)	30 (71)	91.1	71.9	63.3	93.8
7	24 (57)	18 (43)	82.9	82.8	72.0	90.1
8	25 (60)	17 (41)	66.4	89.4	77.0	83.3
9	32 (76)	10 (24)	49.3	95.6	85.7	78.0
10	40 (95)	2 (5)	27.4	99.3	95.2	72.0

eTable 2. Performance of Readers Within the PI-CAI Reader Study for Comparison

Sensitivity, specificity, positive predictive value (PPV) and negative predictive value (NPV) of biparametric MRI assessments of readers participating in the PI-CAI Reader Study (n=400). These performances are evaluated on distinct data from the calibration dataset, yet both datasets exhibit similar cohort characteristics and share a comparable prevalence of clinically significant prostate cancer (csPCa, Gleason Grade group 2). Readers were provided with the same table in the instruction manual.

Reader Operating Point	PI-RADS ≥3	PI-RADS ≥4	PI-RADS ≥5
Sensitivity (%)	89	81	53
Specificity (%)	59	76	91
PPV (%)	53	64	75
NPV(%)	91	89	79

© 2025 Twilt JJ et al. *JAMA Network Open*.

eFigure 3. Reader Characteristics

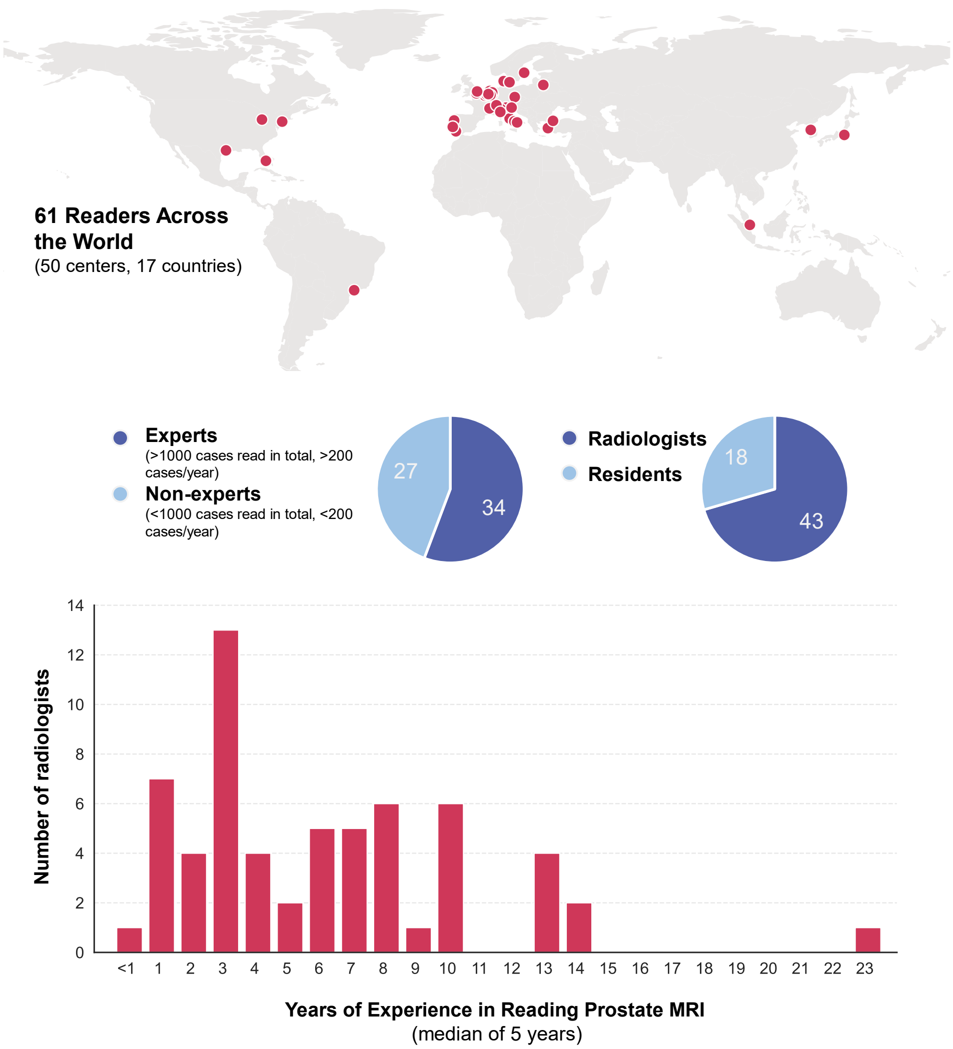

eFigure 3 – Distribution of readers participating in the observer study, including expertise level as per the consensus statements from the European Society of Urogenital Radiology and European Association of Urology [8], and years of experience.

eTable 3. Reader Characteristics per Split-Plot in the Observer Study[a]

	Split A (n = 60)	Split B (n = 60)	Split C (n = 60)	Split D (n = 60)	Split E (n = 60)	Split F (n = 60)
No. readers	11 (18)	8 (13)	10 (16)	11 (18)	11 (18)	10 (16)
Nonexperts	4 (36)	3 (38)	3 (30)	5 (45)	7 (64)	5 (50)
Experts	7 (64)	5 (62)	7 (70)	6 (55)	4 (36)	5 (50)
In Residency	3 (27)	2 (25)	3 (30)	3 (27)	4 (36)	3 (30)
Years of experience[b] (IQR), y	5 (3-9)	6.5 (3-9)	5 (3-8)	4 (3-9)	4 (3-8)	6 (3-8)

Split = Split-plot / sub study; No. = Number; experts = >1000 cases read in total, >200 cases/year [8]; nonexperts = <1000 cases read in total, <200 cases/year [8].
[a] Data are presented as No. (%) unless otherwise indicated
[b] Years of reading prostate MRI

© 2025 Twilt JJ et al. *JAMA Network Open*.

eFigure 4. Schematic Representation of the Reader Study Design

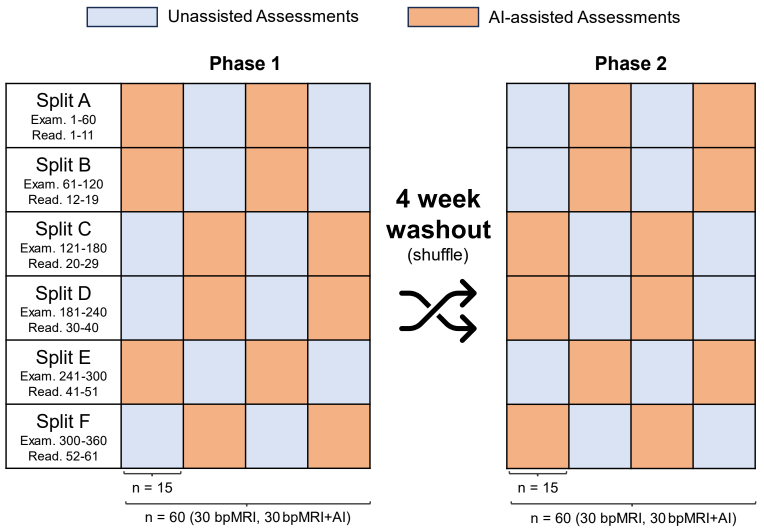

eFigure 4 –Readers (read.) were divided among six reading blocks (split) containing 60/360 examinations (exam.) each. In phase 1, each reader assessed 30/60 biparametric MRI (bpMRI) examinations without AI and 30/60 with AI, in alternating blocks of 15 examinations. Following a 4-week washout period, readers revisited all 60 examinations in phase 2, with the reading configuration reversed from phase 1. The order of examinations was shuffled in phase 2 to reduce recall bias.

eFigure 5. Reader Study Design and Interface

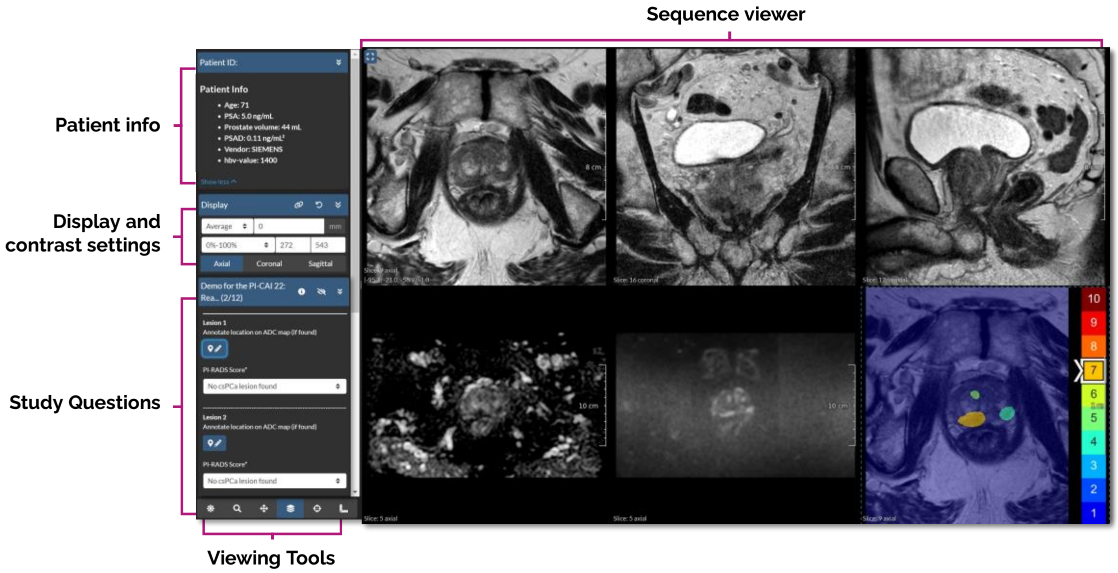

eFigure 5 – Overview of the workstation used to assess biparametric MRI (bpMRI) for detecting clinically significant prostate cancer (csPCa), with and without AI assistance hosted on grand-challenge.org. The sequence viewer displayed the bpMRI imaging protocol (T2-weighted imaging in three planes, axial diffusion-weighted imaging with high b-value images [b ≥ 1000 s/mm²], and the axial apparent diffusion coefficient [ADC] map). The sixth image port (lower right) presented the AI-derived detection map and patient-level score, which were made available during AI-assisted assessments only. The patient info section displayed metadata related to the examination, such as age, PSA level, prostate volume, PSA density, MRI vendor, and high b-value. Remaining sections were used to control settings (display and contrast settings; viewing tools) and answer the study-related questions (study questions).

© 2025 Twilt JJ et al. *JAMA Network Open*.

eAppendix 2. Power Analysis

An a priori power analysis was conducted to determine the necessary number of readers and examinations to achieve a minimum of 80% power for a superiority test comparing unaided versus AI-assisted assessments for diagnosing clinically significant prostate cancer (csPCa) at the patient level. The primary outcome measure was the area under the receiver operating curve (AUROC).

The iMRMC sizing module was used for power calculation, which utilizes the F-test proposed by Hillis et al. [9]. This approach calculates statistical power based on pre-specified multi-reader multi-case (MRMC) components of variance considering the number of readers, split groups, distribution of examinations, the significance level (alpha), and the effect size.

The effect size was set at 0.05, derived from the expected performance difference between biparametric MRI (bpMRI) readings in the PI-CAI Reader Study (AUROC = 0.85) and the standalone performance of top-ranking AI algorithms from PI-CAI (AUROC = 0.90) [1,10]. It was hypothesized that AI assistance would improve reader performance to match that of the standalone AI [11,12]. To estimate MRMC variance components, a reader study simulation was conducted using 20 baseline readings from the PI-CAI Reader Study [1,10]. AI-assisted readings were simulated by adjusting reader scores, targeting an overall AUROC improvement of 0.05. The simulation generated a 95% confidence interval for the pairwise AUROC difference between unaided and AI-assisted reads, ranging from 0.025 to 0.050.

The significance level (alpha) was set at 0.05, and the ratio of negative to positives examinations was set at 70:30, resembling the csPCa prevalence of PI-CAI and large prospective trials [1,10,13,14]. Considering the workload of the radiologists and their willingness to participate, the maximum number of examinations per reader was set at 60 (equals 60 unaided and 60 AI-assisted assessments). Study designs with four to six split plots were considered to ensure sufficient sample sizes for both overall and subgroup analyses [15]. A minimum of 24 readers was expected, with at least six readers within a split plot.

The standard errors and power estimates for various study configurations is shown in eTable 4. A power of 0.8 or greater was achieved with at least 32 readers assessing 240 cases, or 30 readers assessing 320 cases. This power analysis was used to inform the final study design before concluding reader recruitment and examination selection.

eTable 4. Standard Errors and Power Estimates

eTable 4 - Power estimations for study design with total number of examinations of 240, 320 and 360 whilst considering 24 to 56 readers total. The number of cases allocated per reader is read twice: once without AI assistance and once with the support of AI.

Total Cases	Ratio of negative : positive cases	Number of readers	Number of Split Groups	Cases per Reader (total reads)	Readers per Split Group	Total Standard Error	Power
240	70 : 30	24	4	60 (120)	6	0.01808	0.75
240	70 : 30	32	4	60 (120)	8	0.01597	0.86
240	70 : 30	40	4	60 (120)	10	0.01455	0.93
320	70 : 30	30	5	64 (128)	6	0.01578	0.86
320	70 : 30	40	5	64 (128)	8	0.01393	0.95
320	70 : 30	50	5	64 (128)	10	0.01269	0.98
360	70 : 30	36	6	60 (120)	6	0.01477	0.91
360	70 : 30	48	6	60 (120)	8	0.01304	0.97
360	70 : 30	60	6	60 (120)	10	0.01188	0.99

© 2025 Twilt JJ et al. *JAMA Network Open*.

eAppendix 3. Statistical Analysis Plan

As the primary outcome, the diagnostic performance of clinically significant prostate cancer diagnoses is statistically evaluated between the AI-assisted reading arm and the radiologists. Evaluation is performed at a case-level using the area under the receiver operating characteristic curve (AUROC), and sensitivity and specificity at PI-RADS ≥ 3. Empirical receiver operating characteristic (ROC) curves are derived from the case-level suspicion scores (ranging from 0 to 100). Case-level PI-RADS score is defined as the highest PI-RADS score within a case. Multi-reader multi-case analysis of variance (ANOVA) using the Obuchowski-Rockette (OR) (16) method is used to obtain AUROC values, sensitivity and specificity at PI-RADS ≥ 3 operating point and corresponding confidence intervals and mean estimates. To construct the mean AUROC, the ANOVA-OR method estimates the AUROC for each reader, and then calculates the average.

Statistical tests are reserved for primary outcomes, in compliance with expert guidelines. The objectives are prespecified in a hierarchical family to maintain the type-I error for these comparisons and tested accordingly (as shown in eFigure 6) (17). Multiplicity is corrected for using the Holm-Bonferroni method, considering a base alpha value of 0.05. We primarily test for superiority. Superiority of the AI assisted reading arm is concluded if the lower boundary of the two-sided 95%, 97.5% or 98.3% confidence interval (pending correction for multiplicity) for the test statistic is greater than 0. All statistical analyses are done with R version 2022.12.0 software and the MRMCaov package (18-20).

eFigure 6. Objectives for Primary Outcomes

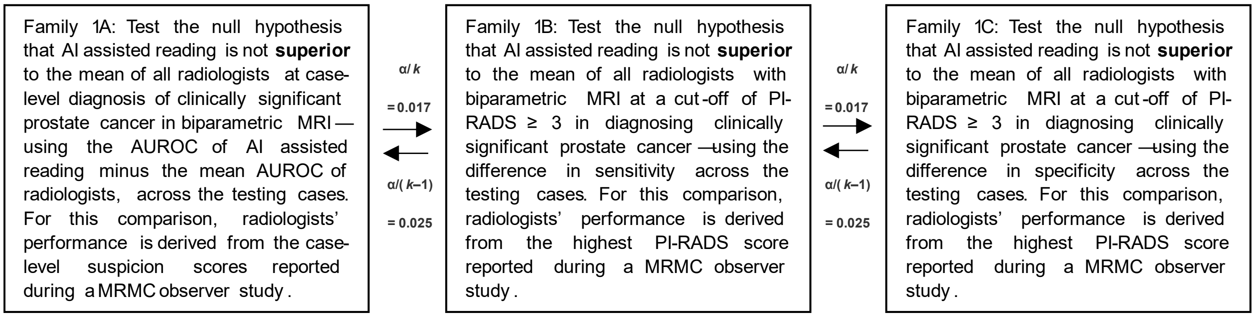

eFigure 6 – Flowchart illustrates the strategic plan to test study objectives while maintaining the type I error rate. Significance thresholds used for family 1A, 1B and 1C are adjusted using the Holm–Bonferroni method, considering a base alpha value of 0.05.

© 2025 Twilt JJ et al. *JAMA Network Open*.

eTable 5. Split-Plot Data and Reader Characteristics[a]

Characteristic	**Split A (n = 60)**	**Split B (n = 60)**	**Split C (n = 60)**	**Split D (n = 60)**	**Split E (n = 60)**	**Split F (n = 60)**
Center						
RUMC	19 (32)	16 (27)	17 (28)	23 (38)	15 (25)	23 (38)
ZGT	14 (23)	19 (32)	13 (22)	17 (28)	19 (32)	15 (25)
PCNN	12 (20)	13 (22)	13 (22)	10 (17)	13 (22)	15 (25)
STOH	15 (25)	12 (20)	17 (28)	10 (17)	13 (22)	7 (12)
Patient Age, median (IQR), y	67 (62, 70)	65 (62, 70)	64 (60, 68)	66 (62, 71)	65 (61, 69)	66 (62, 70)
Patient PSA level, median (IQR), ng/mL	7.1 (5.4, 10.6)	6.8 (4.9, 12.0)	7.0 (5.4, 9.0)	7.9 (5.5, 11.2)	6.4 (5.0, 10.1)	7.6 (5.6, 9.7)
Patient prostate volume, median (IQR), mL	58.0 (41.0, 76.0)	58.0 (42.0, 79.0)	52.0 (38.0, 76.0)	52.0 (38.0, 77.0)	56.0 (42.0, 83.0)	49.0 (38.0, 70.0)
Patient PSAd, median (IQR), ng/mL²	0.12 (0.08, 0.18)	0.13 (0.08, 0.21)	0.13 (0.07, 0.18)	0.14 (0.1, 0.23)	0.12 (0.08, 0.16)	0.16 (0.1, 0.23)
Patient csPCa status						
Without	42 (70)	39 (65)	39 (65)	39 (65)	40 (67)	39 (65)
With	18 (30)	21 (35)	21 (35)	21 (35)	20 (33)	21 (35)
Ground truth verification						
No Bx, follow-up[b]	8 (13)	9 (15)	11 (18)	12 (20)	11 (18)	10 (17)
Sys Bx	25 (42)	20 (33)	18 (30)	19 (32)	24 (40)	20 (33)
MRGBx	12 (20)	7 (12)	8 (13)	8 (13)	5 (8)	7 (12)
Sys Bx and MRGBx	10 (17)	16 (27)	16 (27)	12 (20)	11 (18)	13 (22)
RP	5 (8)	8 (13)	7 (12)	9 (15)	9 (15)	10 (17)
Gleason grade						
0	32 (53)	29 (48)	33 (55)	28 (47)	33 (55)	31 (52)
1	10 (17)	10 (17)	6 (10)	11 (18)	7 (12)	8 (13)
2	6 (10)	11 (18)	13 (22)	7 (12)	12 (20)	8 (13)
3	6 (10)	3 (5)	2 (3)	9 (15)	3 (5)	8 (13)
4	2 (3)	1 (2)	1 (2)	2 (3)	1 (2)	3 (5)
5	4 (7)	6 (10)	5 (8)	3 (5)	4 (7)	2 (3)
PI-RADS score from original report[c]						
1-2	28 (47)	28 (47)	28 (47)	31 (52)	32 (53)	24 (40)
3	5 (8)	2 (3)	3 (5)	4 (7)	8 (13)	5 (8)
4	15 (25)	15 (25)	17 (28)	10 (17)	8 (13)	15 (25)
5	12 (20)	15 (25)	12 (20)	15 (25)	12 (20)	16 (27)
AI Scores						
1	4 (7)	8 (13)	6 (10)	4 (7)	7 (12)	5 (8)
2	6 (10)	6 (10)	9 (15)	6 (10)	11 (18)	12 (20)
3	3 (5)	4 (7)	4 (7)	7 (12)	8 (13)	7 (12)
4	10 (17)	2 (3)	7 (12)	6 (10)	3 (5)	3 (5)
5	11 (18)	9 (15)	9 (15)	8 (13)	8 (13)	4 (7)
6	3 (5)	2 (3)	5 (8)	6 (10)	3 (5)	7 (12)
7	4 (7)	9 (15)	4 (7)	9 (15)	5 (8)	7 (12)
8	4 (7)	7 (12)	6 (10)	3 (5)	1 (2)	6 (10)
9	7 (12)	7 (12)	4 (7)	4 (7)	5 (8)	3 (5)
10	8 (13)	6 (10)	6 (10)	7 (12)	9 (15)	6 (10)

RUMC = Radboud University Medical Center, Netherlands; ZGT = Ziekenhuisgroep Twente, Netherlands; PCNN = Prostaat Centrum Noord-Nederland, Netherlands; STOH = St. Olav's Hospital, Trondheim University Hospital, Norway; IQR = Interquartile range; PSA = Prostate-specific Antigen; PSAd = Prostate-specific Antigen Density; csPCa = Clinically significant prostate cancer (GG≥2); Bx = Biopsy; Sys Bx = Systematic Ultrasound Guided Biopsy; MRGBx = MRI Guided Biopsy; RP = Radical Prostatectomy; PI-RADS = Prostate Imaging Reporting and Data System

[a]Data are presented as No. (%) of patients unless otherwise indicated, [b]Follow-up period of at least 3 years, [c]Defined as the highest score found on a per-patient level as assigned in the original radiology report from routine clinical practice

© 2025 Twilt JJ et al. *JAMA Network Open*.

eTable 6. Calibration Cohort Characteristics[a]

Characteristic	**Total (N = 420)**	**RUMC (n = 175)**	**ZGT (n = 136)**	**PCNN (n = 109)**
Patient Age, median (IQR), y	65 (60, 69)	65 (59, 69)	65 (59, 68)	66 (63, 73)
Patient PSA level, median (IQR), ng/mL	7.2 (5.5, 10.9)	7.1 (5.2, 9.9)	6.6 (5.3, 8.7)	9.2 (6.5, 14.8)
Patient prostate volume, median (IQR), mL	53.0 (40.0, 79.0)	62.0 (45.0, 87.0)	50.0 (40.0, 71.0)	46.0 (33.0, 65.0)
Patient PSAd, median (IQR), ng/mL²	0.14 (0.09, 0.21)	0.11 (0.08, 0.16)	0.14 (0.08, 0.2)	0.19 (0.14, 0.29)
Patient csPCa status				
Without	274 (65)	120 (69)	91 (67)	63 (58)
With	146 (35)	55 (31)	45 (33)	46 (42)
MRI Vendor				
Siemens Healthineers	353 (84)	175 (100)	136 (100)	42 (39)
Philips Medical Systems	67 (16)	0	0	67 (61)
Field strength, T				
1.5	8 (2)	0	0	8 (7)
3	412 (98)	175 (100)	136 (100)	101 (93)
Ground truth verification				
No Bx, follow-up[b]	72 (17)	72 (41)	0	0
Sys Bx	129 (31)	31 (18)	64 (47)	34 (31)
MRGBx	74 (18)	13 (7)	0	61 (56)
Sys Bx and MRGBx	84 (20)	42 (24)	42 (31)	0
RP	61 (15%)	17 (10)	30 (22)	14 (13)
Gleason grade				
0	202 (48)	102 (58)	60 (44)	40 (37)
1	72 (17)	18 (10)	31 (23)	23 (21)
2	78 (19)	26 (15)	30 (22)	22 (20)
3	35 (8)	14 (8)	5 (4)	16 (15)
4	11 (3)	4 (2)	3 (2)	4 (4)
5	22 (5)	11 (6)	7 (5)	4 (4)
PI-RADS score from original report[c]				
1-2	193 (46)	106 (61)	65 (48)	22 (20)
3	24 (6)	6 (3)	6 (4)	12 (11)
4	88 (21)	25 (14)	23 (17)	40 (37)
5	115 (27)	38 (22)	42 (31)	35 (32)
AI Scores				
1	42 (10)	20 (11)	20 (15)	2 (2)
2	42 (10)	20 (11)	16 (12)	6 (6)
3	42 (10)	18 (10)	19 (14)	5 (5)
4	42 (10)	24 (14)	9 (7)	9 (8)
5	42 (10)	17 (10)	14 (10)	11 (10)
6	42 (10)	18 (10)	12 (9)	12 (11)
7	42 (10)	12 (7)	9 (7)	21 (19)
8	42 (10)	14 (8)	10 (7)	18 (17)
9	42 (10)	17 (10)	11 (8)	14 (13)
10	42 (10)	15 (9)	16 (12)	11 (10)

RUMC = Radboud University Medical Center, Netherlands; ZGT = Ziekenhuisgroep Twente, Netherlands; PCNN = Prostaat Centrum Noord-Nederland, Netherlands; STOH = St. Olav's Hospital, Trondheim University Hospital, Norway; IQR = Interquartile range; PSA = Prostate-specific Antigen; PSAd = Prostate-specific Antigen Density; csPCa = Clinically significant prostate cancer (GG≥2); Bx = Biopsy; Sys Bx = Systematic Ultrasound Guided Biopsy; MRGBx = MRI Guided Biopsy; RP = Radical Prostatectomy; PI-RADS = Prostate Imaging Reporting and Data System

[a]Data are presented as No. (%) of patients unless otherwise indicated, [b]Follow-up period of at least 3 years, [c]Defined as the highest score found on a per-patient level as assigned in the original radiology report from routine clinical practice

© 2025 Twilt JJ et al. *JAMA Network Open*.

eFigure 7. Proportion of Diagnoses Across Unassisted and AI-Assisted Assessments Including Expertise Subgroups

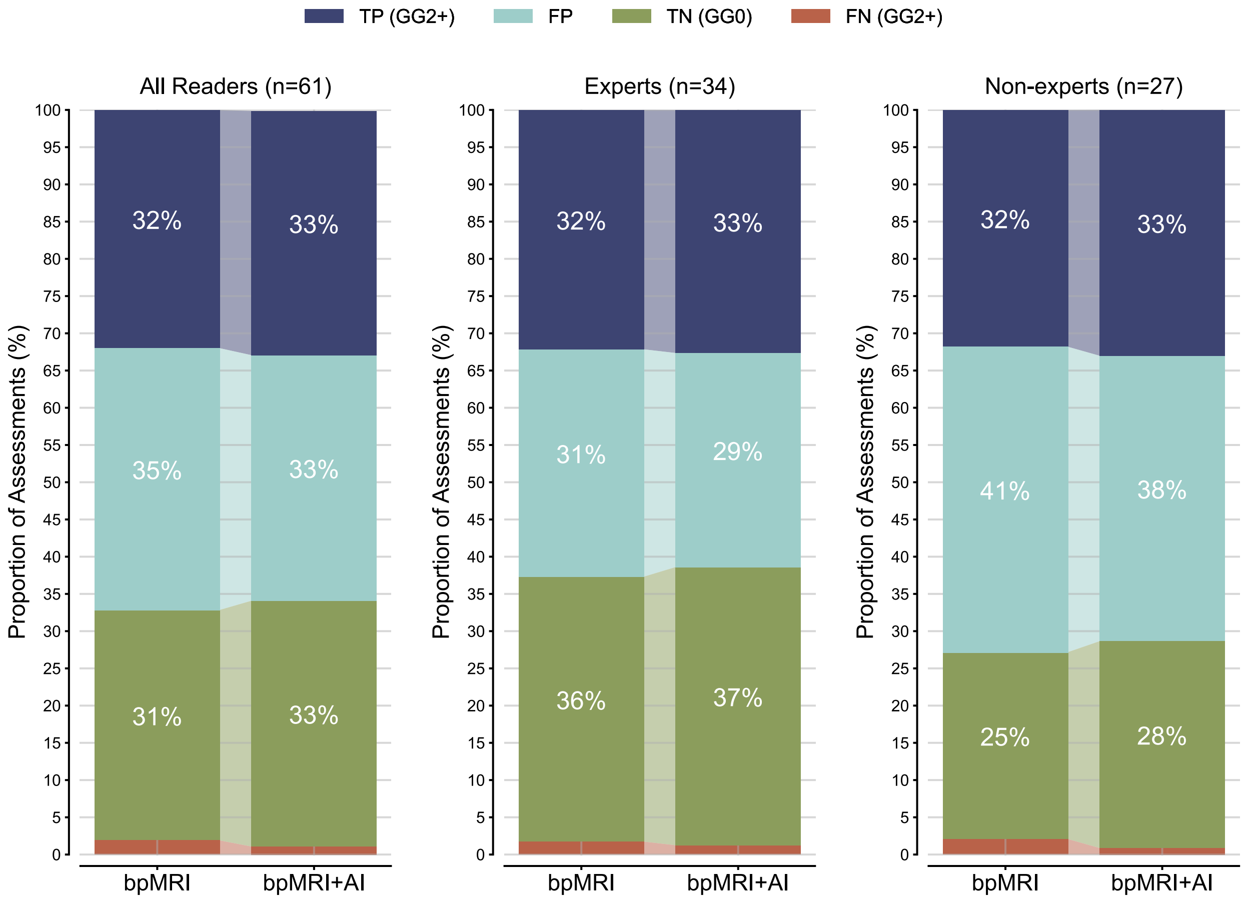

eFigure 7 – The proportion of true positive (TP), false positive (FP), true negative (TN) and false negative (FN) diagnoses for clinically significant prostate cancer, defined as Gleason grade group 2 and higher (GG2+), across unassisted (bpMRI) and AI assisted (bpMRI+AI) assessments. Distributions are shown for all readers and expertise subgroups. AI assistance improved TP and TN diagnoses and reduced the proportion of FP and FN assessments. Nonexperts had a minimal additional benefit of AI across performance metrics compared to experts.

© 2025 Twilt JJ et al. *JAMA Network Open*.

eTable 7. Performance Metrics Across Alternate Operating Points

		bpMRI			bpMRI+AI	
	No. GG1 diagnoses (IQR)	Sensitivity (%) (95% CI)	Specificity (%) (95% CI)	No. GG1 diagnoses (IQR)	Sensitivity (%) (95% CI)	Specificity (%) (95% CI)
PI-RADS ≥4 and PI-RADS 3 with PSAd ≥0.15 ng/ml[2]						
All readers (N = 61)	26 (18-36)	90.9 (87.3-94.5)	66.8 (61.0-72.5)	27 (18-36)	92.9 (89.8-96.1)	69.3 (63.5-76.5)
Experts (n = 34)	26 (18-36)	93.0 (88.9-97.0)	69.1 (61.7-76.5)	29 (18-42)	93.5 (89.9-97.1)	70.2 (63.2-75.0)
Nonexperts (n = 27)	26 (18-30)	88.3 (93.5-93.1)	63.8 (56.3-71.4)	25 (18-30)	92.3 (88.6-95.9)	68.2 (60.1-76.2)
PI-RADS ≥4						
All readers (N = 61)	24 (12-30)	85.7 (81.4-90.1)	72.9 (67.9-77.9)	24 (12-30)	88.7 (84.9-92.6)	75.8 (70.9-80.7)
Experts (n = 34)	25 (18-30)	88.5 (83.4-93.6)	74.3 (67.8-80.8)	25 (18-30)	89.8 (85.5-94.1)	75.9 (70.0-81.7)
Nonexperts (n = 27)	22 (12-24)	82.3 (76.7-87.9)	71.1 (64.3-77.9)	21 (12-30)	87.4 (82.4-92.4)	75.6 (68.9-82.4)

bpMRI = biparametric magnetic resonance imaging; bpMRI+AI = biparametric magnetic resonance imaging with artificial intelligence assistance; No. = number of; 95%CI = 95% Confidence Interval; IQR = Interquartile range; PSAd = Prostate-specific Antigen Density; GG1 = Gleason grade 1, insignificant prostate cancer; PI-RADS = Prostate Imaging Reporting and Data System; experts = >1000 cases read in total and >200 cases/year [8]; nonexperts = <1000 cases read in total and/or <200 cases/year [8].

© 2025 Twilt JJ et al. *JAMA Network Open*.

eFigure 8. Individual Performance Differences of Readers

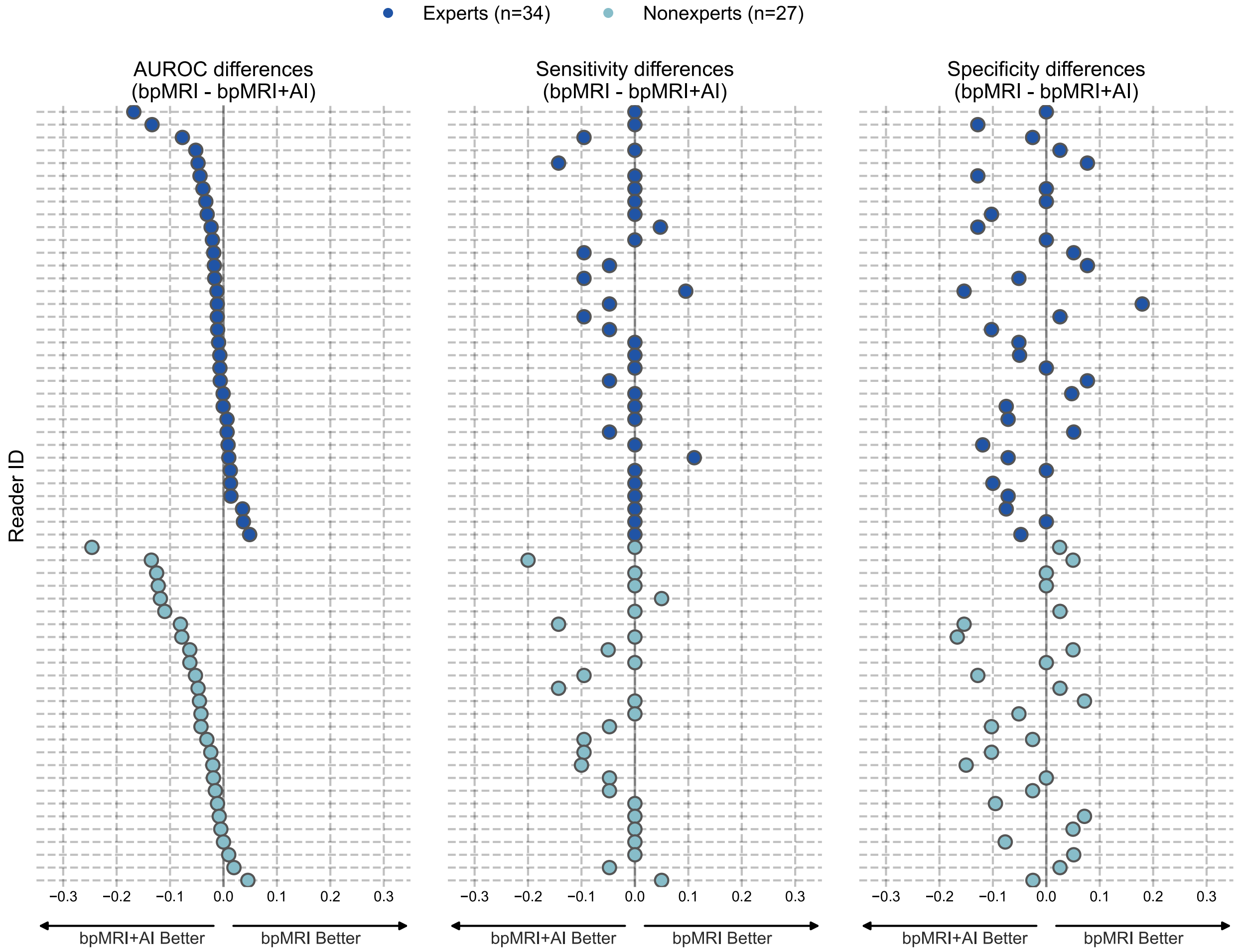

eFigure 8 – Performance differences between biparametric MRI (bpMRI) assessments and bpMRI assessments with AI assistance (bpMRI+AI) for expert readers (n=34) and nonexpert readers (n=27) across all primary endpoints (Area Under the Receiver Operating Curve [AUROC], and sensitivity and specificity at a PI-RADS ≥3 operating point). Expert readers are readers with >1000 cases read in total and >200 cases/year, following 2020 consensus statements from the European Society of Urogenital Radiology and European Association of Urology [8].

© 2025 Twilt JJ et al. *JAMA Network Open*.

eFigure 9. Example of AI-Assisted Upgrading in the Assessment of a Patient With Clinically Significant Prostate Cancer

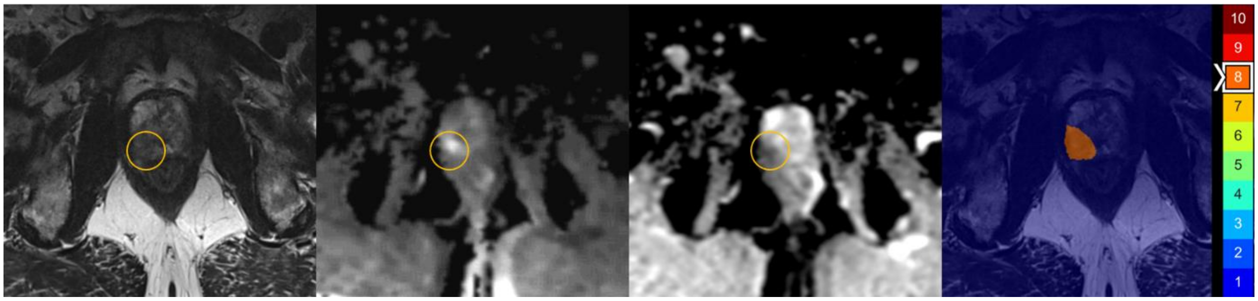

eFigure 9 – Example of AI-assisted upgrading in the assessment of a patient with clinically significant prostate cancer (csPCa, Gleason Grade group 2), with from left to right, axial T2-weighted (T2W), diffusion-weighted imaging (DWI) high b-value, DWI apparent diffusion coefficient (ADC) map, and AI-generated detection map and patient-level score. The patient's prostate-specific antigen (PSA) level was 12.2 ng/mL, with a PSA density (PSAD) of 0.22 ng/mL². A PI-RADS 4 lesion was identified in the right peripheral zone of the apex during clinical routine (yellow). Radical prostatectomy confirmed csPCa (Gleason score 4+3). The AI system scored the examination 8/10. During AI-assisted assessments, four out of eleven readers upgraded their scores from PI-RADS 1-2 to PI-RADS ≥3. Of the remaining readers, four upgraded their PI-RADS ≥3 scores to higher values, two had consistent scores, and one downgraded their score.

eFigure 10. Example of AI-Assisted Downgrading in the Assessment of a Patient With Clinically Significant Prostate Cancer

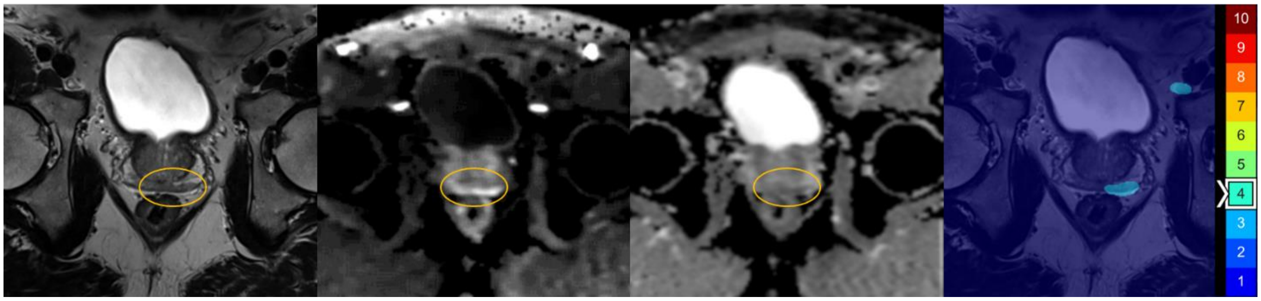

eFigure 10 – Example of AI-assisted downgrading in the assessment of a patient with clinically significant prostate cancer (csPCa, Gleason Grade group 2), with from left to right, axial T2-weighted (T2W), diffusion-weighted imaging (DWI) high b-value, DWI apparent diffusion coefficient (ADC) map, and AI-generated detection map and patient-level score. The patient had a prostate-specific antigen (PSA) level of 5.3 ng/mL and a PSA density (PSAD) of 0.16 ng/mL². During clinical evaluation, a PI-RADS 4 lesion was identified in the left peripheral zone, mid-prostate (yellow). Systematic and targeted biopsies confirmed csPCa (Gleason score 3+4). The AI system scored the examination 4/10, and segmented an area outside the prostate. With AI assistance, three out of ten readers downgraded their scores to PI-RADS 1-2. Four readers had consistent scores, with two initially scoring PI-RADS 1-2. The remaining readers upgraded their scores with AI assistance.

© 2025 Twilt JJ et al. *JAMA Network Open*.

eFigure 11. Example of AI-Assisted Downgrading in the Assessment of a Patient Without Clinically Significant Prostate Cancer

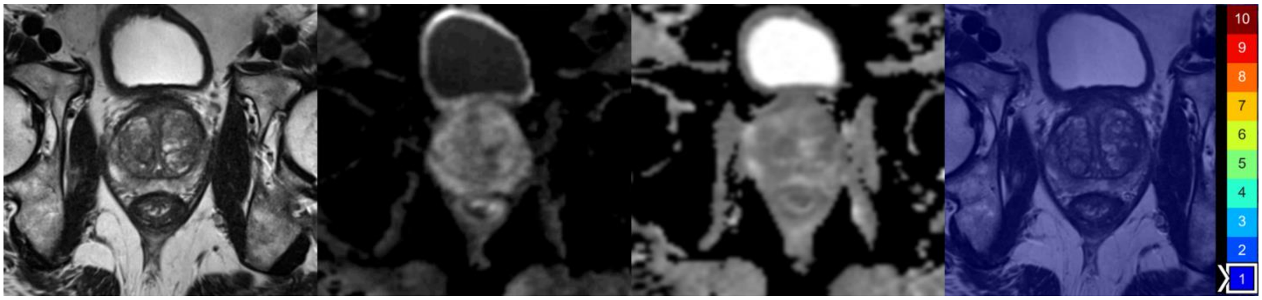

eFigure 11 – Example of AI-assisted downgrading in the assessment of a patient without clinically significant prostate cancer (csPCa, Gleason Grade group 2), with from left to right, axial T2-weighted (T2W), diffusion-weighted imaging (DWI) high b-value, DWI apparent diffusion coefficient (ADC) map and AI-generated detection map and patient-level score. The prostate-specific antigen (PSA) level of this patient was 8.7 ng/mL with a PSA density (PSAD) of 0.10 ng/mL². This patient presented with signs of benign prostatic hyperplasia (BPH) and received a PI-RADS 2 score during clinical routine. Systematic biopsies ruled out the presence of PCa, and the absence of csPCa was confirmed through 65 months of follow-up. The AI system assigned this examination a score of 1/10. With AI assistance, five out of eleven readers downgraded their initial PI-RADS ≥3 assessment to PI-RADS 1-2, eliminating suspicious areas that resembled BPH. The remaining readers either maintained their PI-RADS 1-2 (n=5) or PI-RADS 3 score (n=1).

eFigure 12. Example of AI-Assisted Downgrading in the Assessment of a Patient Without Clinically Significant Prostate Cancer

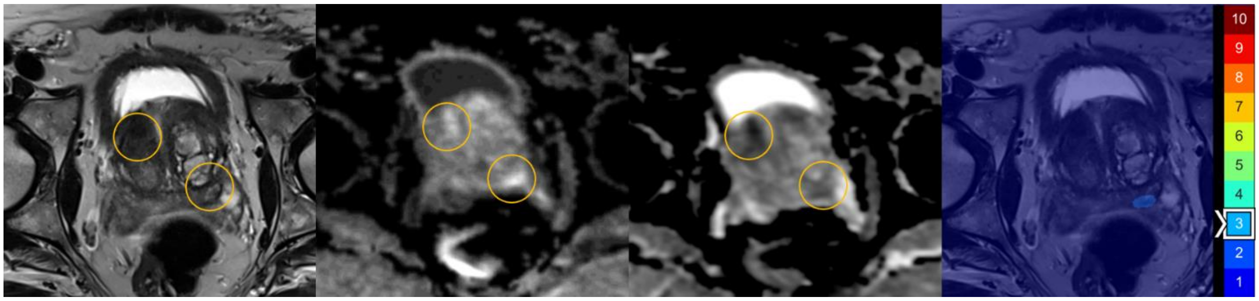

eFigure 12 – Example of AI-assisted downgrading in the assessment of a patient without clinically significant prostate cancer (csPCa, Gleason Grade group 2), with from left to right, axial T2-weighted (T2W), diffusion-weighted imaging (DWI) high b-value, DWI apparent diffusion coefficient (ADC) map and AI-generated detection map and patient-level score. The patient had a prostate-specific antigen (PSA) level of 6.1 ng/mL with a PSA density of 0.07 ng/mL². During clinical routine, PI-RADS 1-2 score was provided. Systematic biopsies were negative for PCa presence. The absence of csPCa was confirmed through 61 months of follow-up. The AI system scored the examination 3/10. During the reader study, two suspicious areas were identified in the peripheral zone and transition zone in the midgland (yellow circles). The area in the right transition zone resembled benign prostatic hyperplasia (BPH), while the area in the left peripheral zone exhibited signs of (post)inflammatory changes or low grade tumor. Overall, five out of eleven readers initially detected one or both of the suspicious areas and provided a patient-level PI-RADS ≥3 score. Upon assessment with AI assistance, they downgraded their scores to PI-RADS 1–2. Among the six remaining readers, three maintained PI-RADS 1-2, two retained PI-RADS 3, and one downgraded their score from PI-RADS 4 to PI-RADS 3.

© 2025 Twilt JJ et al. *JAMA Network Open*.

eFigure 13. Example of AI-Assisted Upgrading in the Assessment of a Patient Without Clinically Significant Prostate Cancer

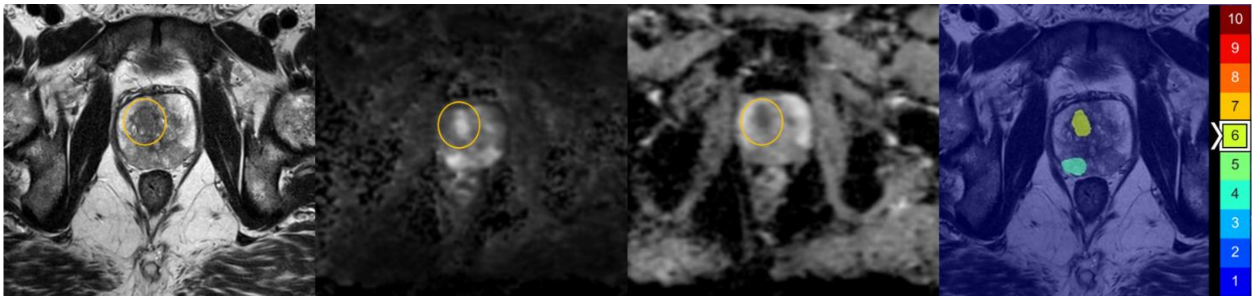

eFigure 13 - Example of AI-assisted upgrading in the assessment of a patient without clinically significant prostate cancer (csPCa, Gleason Grade group 2), with from left to right, axial T2-weighted (T2W), diffusion-weighted imaging (DWI) high b-value, DWI apparent diffusion coefficient (ADC) map and AI-generated detection map and patient-level score. The patient presented with a prostate-specific antigen (PSA) level of 3.7 ng/mL and a PSA density (PSAD) of 0.05 ng/mL². During routine clinical assessment, a PI-RADS 2 score was assigned, indicating benign prostatic hyperplasia (BPH) in the right transition zone (mid/apex) and restrictive diffusion in the right peripheral zone, suggestive of prostatitis. No biopsies were performed, and a 42-month follow-up confirmed the absence of csPCa. The AI system assigned a score of 6/10. With AI assistance, five out of eleven readers upgraded their initial PI-RADS 1-2 scores to PI-RADS ≥3, highlighting one or both of the suspicious areas. Additionally, one reader increased their PI-RADS score from 3 to 4, one maintained a PI-RADS 1-2 classification, and another retained a PI-RADS 5 score. Two readers downgraded from PI-RADS 4 to 3, while one downgraded from PI-RADS 3 to PI-RADS 1-2.

eFigure 14. Example of AI-Assisted Upgrading in the Assessment of a Patient Without Clinically Significant Prostate Cancer

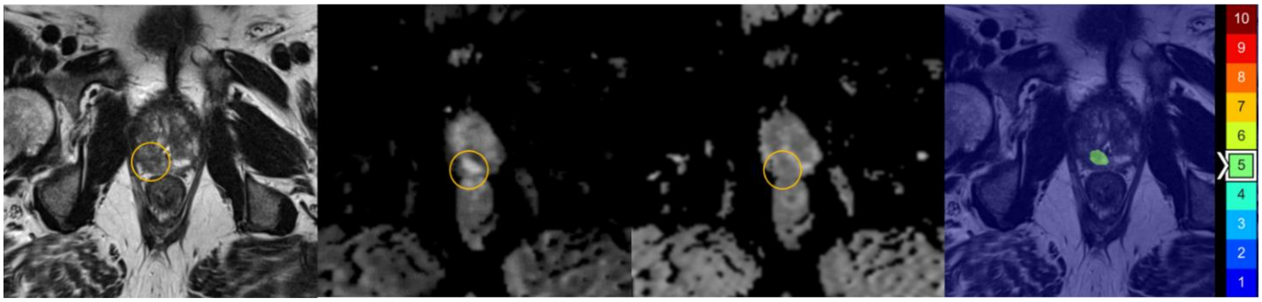

eFigure 14 – Example of AI-assisted upgrading in the assessment of a patient without clinically significant prostate cancer (csPCa, Gleason Grade group 2), with from left to right, axial T2-weighted (T2W), diffusion-weighted imaging (DWI) high b-value, DWI apparent diffusion coefficient (ADC) map and AI-generated detection map and patient-level score. The patient presented with a prostate-specific antigen (PSA) level of 9 ng/mL and a PSA density (PSAD) of 0.10 ng/mL². In clinical routine, a PI-RADS 4 lesion (yellow) was identified in the right peripheral zone at the apex. Systematic and targeted biopsies confirmed the absence of csPCa. Following AI assistance, five out of eleven readers upgraded their initial assessments from PI-RADS 1-2 to PI-RADS ≥3, and one reader upgraded from PI-RADS 3 to PI-RADS 4. The remaining readers either maintained their original scores (n=3) or downgraded their assessments (n=2).

© 2025 Twilt JJ et al. *JAMA Network Open*.

eFigure 15. Proportion of PI-RADS Scores Across Unassisted and AI-Assisted Assessments for Expertise Subgroups

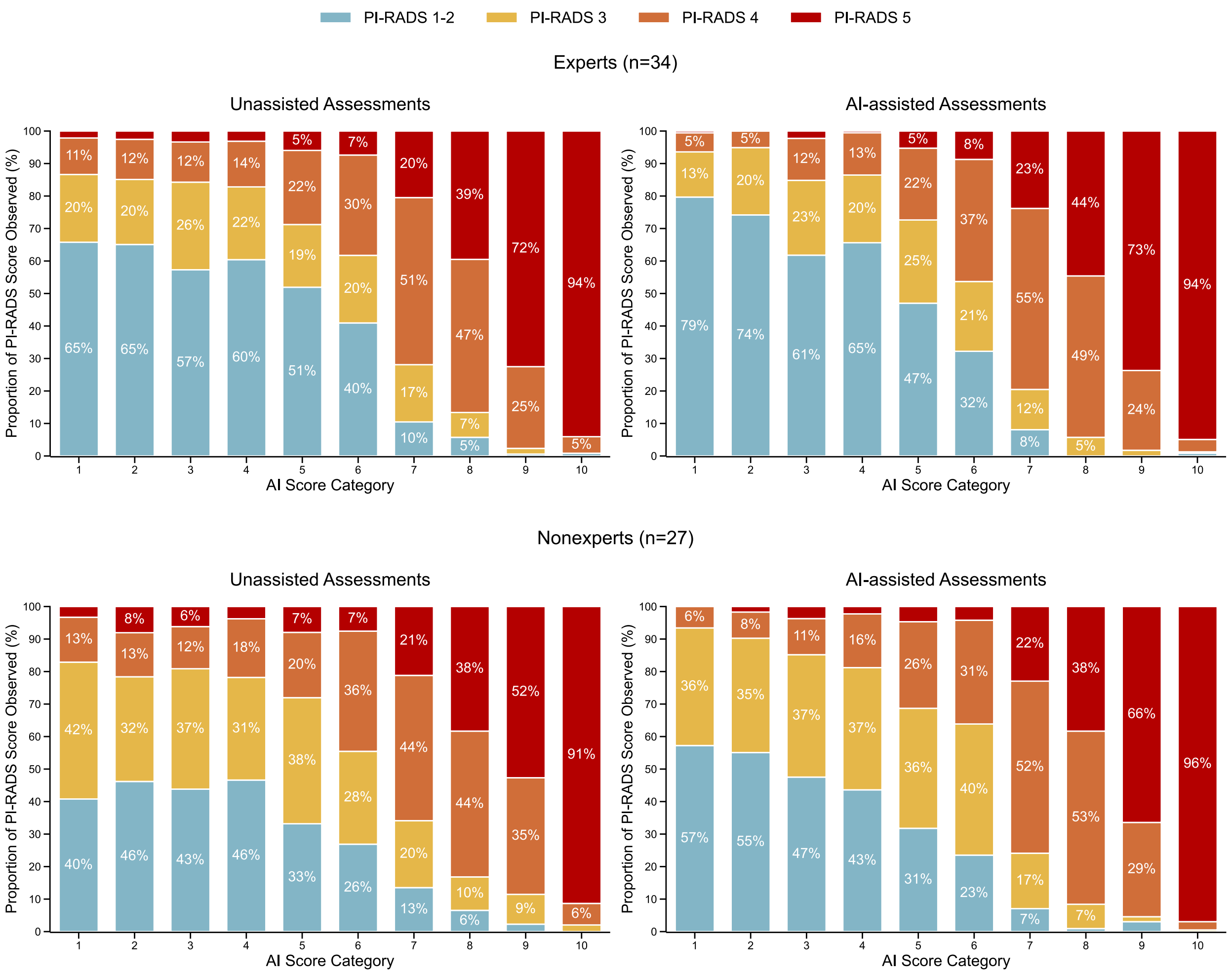

eFigure 15 – Proportion of PI-RADS score observed for unassisted (left) and AI-assisted (right) assessments for experts (n=34) (top) and nonexpert readers (n=27) (bottom), grouping examinations according to their AI score. For both reader groups, AI assistance resulted in a higher proportion of PI-RADS 1-2 scores in lower AI score categories and a reduced proportion in higher categories. Conversely, PI-RADS 4-5 scores decreased in lower categories and increased in higher categories with AI assistance. Overall, experts had lower PI-RADS 3 scores across all AI score categories.

© 2025 Twilt JJ et al. *JAMA Network Open*.

eReferences

1. Saha A, Bosma JS, Twilt JJ, et al. Artificial intelligence and radiologists in prostate cancer detection on MRI (PI-CAI): an international, paired, non-inferiority, confirmatory study. Lancet Oncol. 2024;25(7):879-887.
2. Debs N, Routier A, Abi-Nader C, et al. Deep learning for detection and diagnosis of prostate cancer from bpMRI and PSA: Guerbet's contribution to the PI-CAI 2022 Grand Challenge. 11 October 2024. https://pi-cai.grand-challenge.org/algorithms/
3. Yuan Y, Ahn E, Feng D, et al. Z-SSMNet: A Zonal-aware Self-Supervised Mesh Network for Prostate Cancer Detection and Diagnosis in bpMRI. 11 October 2024. https://pi-cai.grand-challenge.org/algorithms/
4. Kan H, Qiao L, Shi J, et al. Implementation method of the PI-CAI Challenge (Swangeese Team). 11 October 2024. https://pi-cai.grand-challenge.org/algorithms/
5. Li X, Vesal S, Saunders S, et al. The Prostate Imaging: Cancer AI (PI-CAI) 2022 Grand Challenge (PIMed Team). 11 October 2024. https://pi-cai.grand-challenge.org/algorithms/
6. Karagöz A, Şeker ME, Yergin M, et al. Prostate Lesion Estimation using Prostate Masks from Biparametric MRI. 11 October 2024. https://pi-cai.grand-challenge.org/algorithms/
7. Bosma JS, Saha A, Hosseinzadeh M, et al. Semisupervised Learning with Report-guided Pseudo Labels for Deep Learning–based Prostate Cancer Detection Using Biparametric MRI. Radiol Artif Intell. 2023;5(5).
8. de Rooij M, Israël B, Tummers M, et al. ESUR/ESUI consensus statements on multi-parametric MRI for the detection of clinically significant prostate cancer: quality requirements for image acquisition, interpretation and radiologists' training. European Radiology. 2020;30(10):5404.
9. Hillis SL, Obuchowski NA, Berbaum KS. Power Estimation for Multireader ROC Methods. An Updated and Unified Approach. Acad. Radiol. 2011;18(2):129-142.
10. Twilt JJ, Saha A, Bosma JS, van Ginneken B, Bjartell A, Padhani AR, et al. Evaluating Biparametric Versus Multiparametric Magnetic Resonance Imaging for Diagnosing Clinically Significant Prostate Cancer: An International, Paired, Noninferiority, Confirmatory Observer Study. Eur Urol. 2024;0(0).
11. Sun Z, Wang K, Kong Z, et al. A multicenter study of artificial intelligence-aided software for detecting visible clinically significant prostate cancer on mpMRI. Insights into Imaging. 2023;14(1).
12. Labus S, Altmann MM, Huisman H, et al. A concurrent, deep learning–based computer-aided detection system for prostate multiparametric MRI: a performance study involving experienced and less-experienced radiologists. Eur. Radiol. 2023;33(1):64-76.
13. van der Leest M, Cornel E, Israël B, et al. Head-to-head Comparison of Transrectal Ultrasound-guided Prostate Biopsy Versus Multiparametric Prostate Resonance Imaging with Subsequent Magnetic Resonance-guided Biopsy in Biopsy-naïve Men with Elevated Prostate-specific Antigen: A Large Prospective Multicenter Clinical Study. Eur. Urol. 2019;75(4):570-578.
14. Ahmed HU, El-Shater Bosaily A, Brown LC, et al. Diagnostic accuracy of multi-parametric MRI and TRUS biopsy in prostate cancer (PROMIS): a paired validating confirmatory study. Lancet. 2017;25;389(10071):815-822.
15. Chen W, Gong Q, Gallas BD. Paired split-plot designs of multireader multicase studies. Journal of Medical Imaging. 2018;5(3):1.
16. Obuchowski NA, Rockette HE. Hypothesis testing of diagnostic accuracy for multiple readers and multiple tests: an ANOVA approach with dependent observations. Communications in Statistics-Simulation and Computation 1995; 24(2), 285-308.
17. Obuchowski NA, Bullen J. Multireader diagnostic accuracy imaging studies: fundamentals of design and analysis. Radiology 2022; 303(1): 26-34
18. R Core Team (2022). R: A language and environment for statistical computing. R Foundation for Statistical Computing, Vienna, Austria. URL https://www.R-project.org/.
19. Smith BJ, Hillis SL. Multi-reader multi-case analysis of variance software for diagnostic performance comparison of imaging modalities. Proc SPIE Int Soc Opt Eng. 2020; 11316:113160K.
20. Smith BJ, Hillis SL, Pesce LL. MCMCaov: Multi-Reader Multi-Case Analysis of Variance. Version 0.3.0. 2023. Available from: https://cran.r-project.org/package=MRMCaov.

© 2025 Twilt JJ et al. *JAMA Network Open*.
