# Supplementary material for: AI-Assisted vs Unassisted Identification of Prostate Cancer in Magnetic Resonance Images
Source: JAMA Netw Open. 2025 Jun 13;8(6):e2515672. doi: 10.1001/jamanetworkopen.2025.15672 (PMC12166490; doi:10.1001/jamanetworkopen.2025.15672)
Supplement: Supplement 2. — Nonauthor Collaborators [file jamanetwopen-e2515672-s002.pdf]

Supplemental Online Content: Nonauthor Collaborators
*First name, last name, and suffix (if applicable) are required and will appear in PubMed.

*Group Name(s): PI-CAI Consortium							
***First Name and Middle Initial(s)**	***Last Name**	***Suffix (eg, Jr, III)**	Academic Degrees	Institution	Location (city, state/province, country)	Role or Contribution, eg, chair, principal investigator	Group (if more than 1 Group listed in the byline) and/or Subgroup (eg, Steering Committee)
Jasper J.	Twilt		M.Sc.	Radboud University Medical Center	The Netherlands		PI-CAI Consortium, Investigators
Anindo	Saha		M.Sc.	Radboud University Medical Center	The Netherlands		PI-CAI Consortium, Investigators
Joeran S.	Bosma		M.Sc.	Radboud University Medical Center	The Netherlands		PI-CAI Consortium, Investigators
Derya	Yakar		M.D.	Netherlands Cancer Institute; University Medical Center Groningen	The Netherlands		PI-CAI Consortium, Investigators
Mattijs	Elschot		Ph.D.	Norwegian University of Science and Technology	Norway		PI-CAI Consortium, Investigators
Jeroen	Veltman		M.D.	Ziekenhuis Groep Twente; University of Twente	The Netherlands		PI-CAI Consortium, Investigators
Jurgen J.	Fütterer		M.D.	Radboud University Medical Center	The Netherlands		PI-CAI Consortium, Investigators
Maarten	de Rooij		M.D.	Radboud University Medical Center	The Netherlands		PI-CAI Consortium, Investigators
Henkjan	Huisman		Ph.D.	Radboud University Medical Center; Norwegian University of Science and Technology	The Netherlands; Norway		PI-CAI Consortium, Investigators
Anders	Bjartell		M.D.	Skåne University Hospital and Lund University	Sweden		PI-CAI Consortium, Scientific Advisory Board
Anwar R.	Padhani		M.D.	Mount Vernon Cancer Centre	United Kingdom		PI-CAI Consortium, Scientific Advisory Board
David	Bonekamp		M.D.	Deutsches Krebsforschungszentrum Heidelberg	Germany		PI-CAI Consortium, Scientific Advisory Board
Geert	Villeirs		M.D.	Ghent University Hospital	Belgium		PI-CAI Consortium, Scientific Advisory Board

Supplemental Online Content: Nonauthor Collaborators
*First name, last name, and suffix (if applicable) are required and will appear in PubMed.

***First Name and Middle Initial(s)**	***Last Name**	***Suffix (eg, Jr, III)**	Academic Degrees	Institution	Location (city, state/province, country)	Role or Contribution, eg, chair, principal investigator	Group (if more than 1 Group listed in the byline) and/or Subgroup (eg, Steering Committee)
Georg	Salomon		M.D.	University Hospital Hamburg-Eppendorf	Germany		PI-CAI Consortium, Scientific Advisory Board
Gianluca	Giannarini		M.D.	Santa Maria della Misericordia University Hospital	Udine		PI-CAI Consortium, Scientific Advisory Board
Henkjan	Huisman		Ph.D.	Radboud University Medical Center; Norwegian University of Science and Technology	The Netherlands; Norway		PI-CAI Consortium, Scientific Advisory Board
Jayashree	Kalpathy-Cramer		Ph.D.	University of Colorado	United States		PI-CAI Consortium, Scientific Advisory Board
Jelle	Barentsz		M.D.	Andros Clinics	The Netherlands		PI-CAI Consortium, Scientific Advisory Board
Klaus H.	Maier-Hein		Ph.D.	Heidelberg University Hospital; Deutsches Krebsforschungszentrum Heidelberg	Germany		PI-CAI Consortium, Scientific Advisory Board
Mattijs	Elschot		Ph.D.	Norwegian University of Science and Technology	St. Olavs Hospital		PI-CAI Consortium, Scientific Advisory Board
Mirabela	Rusu		Ph.D.	Stanford University	United States		PI-CAI Consortium, Scientific Advisory Board
Nancy A.	Obuchowski		Ph.D.	Cleveland Clinic Foundation	United States		PI-CAI Consortium, Scientific Advisory Board
Olivier	Rouviere		M.D.	Hospices Civils de Lyon	France		PI-CAI Consortium, Scientific Advisory Board
Roderick	van den Bergh		M.D.	Erasmus Medical Center; Saint Antonius Hospital	The Netherlands		PI-CAI Consortium, Scientific Advisory Board

Supplemental Online Content: Nonauthor Collaborators
*First name, last name, and suffix (if applicable) are required and will appear in PubMed.

***First Name and Middle Initial(s)**	***Last Name**	***Suffix (eg, Jr, III)**	Academic Degrees	Institution	Location (city, state/province, country)	Role or Contribution, eg, chair, principal investigator	Group (if more than 1 Group listed in the byline) and/or Subgroup (eg, Steering Committee)
Valeria	Panebianco		M.D.	Sapienza University of Rome	Italy		PI-CAI Consortium, Scientific Advisory Board
Veeru	Kasivisvanathan		M.D.	University College London; University College London Hospital	United Kingdom		PI-CAI Consortium, Scientific Advisory Board
Afsoun	Malakoti-Fard		M.D.	Denmark University Hospital Herlev	Denmark		PI-CAI Consortium, Readers participating in the observer study
Ailin	Dehghanpour		M.D.	Sapienza Univeristy of Rome	Italy		PI-CAI Consortium, Readers participating in the observer study
Ana Sofia L.	Moreira		M.D.	Unidade Local de Saúde Algarve	Portugal		PI-CAI Consortium, Readers participating in the observer study
Andrea	Cazzato		M.D.	University of Genova	Italy		PI-CAI Consortium, Readers participating in the observer study
Andrea	Ponsiglione		M.D.	University of Naples Federico II	Italy		PI-CAI Consortium, Readers participating in the observer study
Arnaldo	Stanzione		M.D.	University of Naples Federico II	Italy		PI-CAI Consortium, Readers participating in the observer study
Bart	de Keyzer		M.D.	Sint Trudo Hospital	Belgium		PI-CAI Consortium, Readers participating in the observer study
Bodil G.	Pedersen		M.D.	Aarhus University Hospital	Denmark		PI-CAI Consortium, Readers participating in the observer study
Christopher	Page		M.D.	Chelsea and Westminster NHS	United Kingdom		PI-CAI Consortium, Readers participating in the observer study

Supplemental Online Content: Nonauthor Collaborators
*First name, last name, and suffix (if applicable) are required and will appear in PubMed.

***First Name and Middle Initial(s)**	***Last Name**	***Suffix (eg, Jr, III)**	Academic Degrees	Institution	Location (city, state/province, country)	Role or Contribution, eg, chair, principal investigator	Group (if more than 1 Group listed in the byline) and/or Subgroup (eg, Steering Committee)
Cindy	Mai		M.D.	University Hospital Antwerp	Belgium		PI-CAI Consortium, Readers participating in the observer study
Deniz C.	Alis		M.D.	Acibadem Mehmet Ali Aydnlar University, School of Medicine	Turkey		PI-CAI Consortium, Readers participating in the observer study
Dirk	Versteegden		M.D.	Radboud University Medical Center	The Netherlands		PI-CAI Consortium, Readers participating in the observer study
Enrico	Camisassa		M.D.	IRCCS Ospedale San Raffaele	Italy		PI-CAI Consortium, Readers participating in the observer study
Ewout C.	Staal		M.D.	Maasstad Ziekenhuis	The Netherlands		PI-CAI Consortium, Readers participating in the observer study
Federica	Martini		M.D.	Ospedale Santa Chiara Trento	Italy		PI-CAI Consortium, Readers participating in the observer study
Francesco	Alessandrino		M.D.	University of Miami	United States		PI-CAI Consortium, Readers participating in the observer study
Fredrik	Jäderling		M.D.	Karolinska Institutet	Sweden		PI-CAI Consortium, Readers participating in the observer study
Georgios	Agrotis		M.D.	Netherlands Cancer Institute	The Netherlands		PI-CAI Consortium, Readers participating in the observer study
Giacomo	Avesani		M.D.	Fondazione Policlinico Universitario A. Gemelli IRCCS	Italy		PI-CAI Consortium, Readers participating in the observer study
Giorgio	Brembilla		M.D.	IRCCS Ospedale San Raffaele	Italy		PI-CAI Consortium, Readers participating in the observer study

Supplemental Online Content: Nonauthor Collaborators
*First name, last name, and suffix (if applicable) are required and will appear in PubMed.

*First Name and Middle Initial(s)	*Last Name	*Suffix (eg, Jr, III)	Academic Degrees	Institution	Location (city, state/province, country)	Role or Contribution, eg, chair, principal investigator	Group (if more than 1 Group listed in the byline) and/or Subgroup (eg, Steering Committee)
Giulia	Francese		M.D.	IRCCS Ospedale Policlinico San Martino	Italy		PI-CAI Consortium, Readers participating in the observer study
Henricus P.J. Raat	Raat		M.D.	Laurentius Hospital Roermond	The Netherlands		PI-CAI Consortium, Readers participating in the observer study
Hilal	Sahin		M.D.	University of Health Sciences, Tepecik Training and Research Hospital	Turkey		PI-CAI Consortium, Readers participating in the observer study
Ivo	Schoots		M.D.	Erasmus University Medical Center	The Netherlands		PI-CAI Consortium, Readers participating in the observer study
Iztok	Caglic		M.D.	Cambridge University Hospitals and University of Cambridge	United Kingdom		PI-CAI Consortium, Readers participating in the observer study
Jeries P.	Zawaideh		M.D.	IRCCS Ospedale Policlinico San Martino	Italy		PI-CAI Consortium, Readers participating in the observer study
Leonardo K.	Bittencourt		M.D.	University Hospitals & Case Western Reserve University	United States		PI-CAI Consortium, Readers participating in the observer study
Luigi	Mannacio		M.D.	University of Naples Federico II	Italy		PI-CAI Consortium, Readers participating in the observer study
Matilde L.R.R.	Gonçalves		M.D.	Unidade Local de Saúde Algarve - Faro	Portugal		PI-CAI Consortium, Readers participating in the observer study
Merve Ş.	Özdemir		M.D.	Basaksehir Cam and Sakura City Hospital	Turkey		PI-CAI Consortium, Readers participating in the observer study
Michael	Nahouraii		M.D.	University of Miami	United States		PI-CAI Consortium, Readers participating in the observer study

Supplemental Online Content: Nonauthor Collaborators
*First name, last name, and suffix (if applicable) are required and will appear in PubMed.

***First Name and Middle Initial(s)**	***Last Name**	***Suffix (eg, Jr, III)**	Academic Degrees	Institution	Location (city, state/province, country)	Role or Contribution, eg, chair, principal investigator	Group (if more than 1 Group listed in the byline) and/or Subgroup (eg, Steering Committee)
Miguel N.C.	da Silva		M.D.	Centro Hospitalar Universitário de São João	Portugal		PI-CAI Consortium, Readers participating in the observer study
Misbah	Khurram		M.D.	Herlev and Gentofte Hospital Copenhagen	Denmark		PI-CAI Consortium, Readers participating in the observer study
Moon Hyung	Choi		M.D.	Eunpyeong St. Mary's Hospital; The Catholic University of Korea	South Korea		PI-CAI Consortium, Readers participating in the observer study
Olivier	Rouviere		M.D.	Hospices Civils de Lyon	France		PI-CAI Consortium, Readers participating in the observer study
Paolo N.	Franco		M.D.	Fondazione IRCCS San Gerardo	Italy		PI-CAI Consortium, Readers participating in the observer study
Paulo S.	Correia		M.D.	Unidade Local de Saúde de São José	Portugal		PI-CAI Consortium, Readers participating in the observer study
Pedro R.R.	Riesenberger		M.D.	Unidade Local de Saúde de São José	Portugal		PI-CAI Consortium, Readers participating in the observer study
Petr	Hanus		M.D.	First Faculty of Medicine Charles University	Czech Republic		PI-CAI Consortium, Readers participating in the observer study
Pieter	de Visschere		M.D.	Ghent University Hospital	Belgium		PI-CAI Consortium, Readers participating in the observer study
Ramette Guillaume	Guillaume		M.D.	CHU Lille	France		PI-CAI Consortium, Readers participating in the observer study
Renato	Cuocolo		M.D.	University of Salerno	Italy		PI-CAI Consortium, Readers participating in the observer study

Supplemental Online Content: Nonauthor Collaborators
*First name, last name, and suffix (if applicable) are required and will appear in PubMed.

***First Name and Middle Initial(s)**	***Last Name**	***Suffix (eg, Jr, III)**	Academic Degrees	Institution	Location (city, state/province, country)	Role or Contribution, eg, chair, principal investigator	Group (if more than 1 Group listed in the byline) and/or Subgroup (eg, Steering Committee)
Ricardo O.	Falcão		M.D.	Grupo Aliança	Brazil		PI-CAI Consortium, Readers participating in the observer study
Rogier S.A.	van Stiphout		M.D.	Laurentius Hospital Roermond	The Netherlands		PI-CAI Consortium, Readers participating in the observer study
Rossano	Girometti		M.D.	University of Udine	Italy		PI-CAI Consortium, Readers participating in the observer study
Rossi	Gabriele		M.D.	IRCCS Ospedale Policlinico San Martino	Italy		PI-CAI Consortium, Readers participating in the observer study
Ruta Briediene	Briediene		M.D.	Affidea, National Cancer Institute Lithuania	Lithuania		PI-CAI Consortium, Readers participating in the observer study
Rūta	Grigienė		M.D.	Affidea, National Cancer Institute Lithuania	Lithuania		PI-CAI Consortium, Readers participating in the observer study
Samuel	Withey		M.D.	Royal Marsden Hospital	United Kingdom		PI-CAI Consortium, Readers participating in the observer study
Selahattin	Durmaz		M.D.	Gaziosmanpasa Training and Research Hospital	Turkey		PI-CAI Consortium, Readers participating in the observer study
Sofia F.	Santos		M.D.	Portuguese Oncology Institute, Instituto Português de Oncologia de Lisboa Francisco Gentil	Portugal		PI-CAI Consortium, Readers participating in the observer study
Tommaso	Russo		M.D.	IRCCS Ospedale San Raffaele	Italy		PI-CAI Consortium, Readers participating in the observer study
Tristan	Barrett		M.D.	University of Cambridge	United Kingdom		PI-CAI Consortium, Readers participating in the observer study

*First name, last name, and suffix (if applicable) are required and will appear in PubMed.

***First Name and Middle Initial(s)**	***Last Name**	***Suffix (eg, Jr, III)**	Academic Degrees	Institution	Location (city, state/province, country)	Role or Contribution, eg, chair, principal investigator	Group (if more than 1 Group listed in the byline) and/or Subgroup (eg, Steering Committee)
Valerio	Forte		M.D.	San Carlo di Nancy Hospital	Italy		PI-CAI Consortium, Readers participating in the observer study
Varaha S.	Tammisetti		M.D.	University of Texas McGovern Medical School	United States		PI-CAI Consortium, Readers participating in the observer study
Verena C.	Obmann		M.D.	University of Bern, Department of Diagnostic, Interventional and Pediatric Radiology, Inselspital, Bern; Switzerland and Zuger Kantonsspital, Baar	Switzerland		PI-CAI Consortium, Readers participating in the observer study
William	Weston		M.D.	University College London	United Kingdom		PI-CAI Consortium, Readers participating in the observer study
Yan Mee	Law		M.D.	Singapore General Hospital	Singapore		PI-CAI Consortium, Readers participating in the observer study
Yesim Y.	Yuruk		M.D.	University of Health Sciences, Tepecik Training and Research Hospital	Turkey		PI-CAI Consortium, Readers participating in the observer study
Yu-Cherng	Chang		M.D.	Jackson Memorial Hospital	United States		PI-CAI Consortium, Readers participating in the observer study
Yuki	Arita		M.D.	Memorial Sloan Kettering Cancer Center; Keio University	United States; Japan		PI-CAI Consortium, Readers participating in the observer study
