# Supplementary material for: AI-Assisted vs Unassisted Identification of Prostate Cancer in Magnetic Resonance Images
Source: JAMA Netw Open. 2025 Jun 13;8(6):e2515672. doi: 10.1001/jamanetworkopen.2025.15672 (PMC12166490; doi:10.1001/jamanetworkopen.2025.15672)
Supplement: Supplement 3. — Data Sharing Statement [file jamanetwopen-e2515672-s003.pdf]

Data Sharing Statement

Twilt. AI-Assisted vs Unassisted Identification of Prostate Cancer in Magnetic Resonance Images. *JAMA Netw Open*. Published June 13, 2025.
doi:10.1001/jamanetworkopen.2025.15672

Data
Data available: No
